# Genome-wide meta-analysis of iron status biomarkers and the effect of iron on all-cause mortality in HUNT

**DOI:** 10.1038/s42003-022-03529-z

**Published:** 2022-06-16

**Authors:** Marta R. Moksnes, Sarah E. Graham, Kuan-Han Wu, Ailin Falkmo Hansen, Sarah A. Gagliano Taliun, Wei Zhou, Ketil Thorstensen, Lars G. Fritsche, Dipender Gill, Amy Mason, Francesco Cucca, David Schlessinger, Gonçalo R. Abecasis, Stephen Burgess, Bjørn Olav Åsvold, Jonas B. Nielsen, Kristian Hveem, Cristen J. Willer, Ben M. Brumpton

**Affiliations:** 1 K.G. Jebsen Center for Genetic Epidemiology, Department of Public Health and Nursing, NTNU – Norwegian University of Science and Technology, Trondheim, Norway; 2 Division of Cardiovascular Medicine, Department of Internal Medicine, University of Michigan, Ann Arbor, MI USA; 3 Department of Computational Medicine and Bioinformatics, University of Michigan, Ann Arbor, MI USA; 4 Department of Medicine and Department of Neurosciences, Université de Montréal, Montréal, QC Canada; 5 Montréal Heart Institute, Montréal, QC Canada; 6 Analytic and Translational Genetics Unit, Department of Medicine, Massachusetts General Hospital, Boston, MA USA; 7 Stanley Center for Psychiatric Research, Broad Institute of MIT and Harvard, Cambridge, MA USA; 8 Department of Clinical Chemistry, St. Olavs hospital Trondheim University Hospital, Trondheim, Norway; 9 Department of Biostatistics, University of Michigan School of Public Health, Ann Arbor, MI USA; 10 Center for Statistical Genetics, University of Michigan School of Public Health, Ann Arbor, MI USA; 11 Department of Epidemiology and Biostatistics, School of Public Health, Imperial College London, London, UK; 12 Clinical Pharmacology and Therapeutics Section, Institute for Infection and Immunity, St George’s, University of London, London, UK; 13 Clinical Pharmacology Group, Pharmacy and Medicines Directorate, St George’s University Hospitals NHS Foundation Trust, London, UK; 14 Novo Nordisk Research Centre Oxford, Old Road Campus, Oxford, UK; 15 British Heart Foundation Cardiovascular Epidemiology Unit, Department of Public Health and Primary Care, University of Cambridge, Cambridge, UK; 16 Istituto di Ricerca Genetica e Biomedica, Consiglio Nazionale delle Ricerche (CNR), Cagliari, Italy; 17 Dipartimento di Scienze Biomediche, Università degli Studi di Sassari, Sassari, Italy; 18 Laboratory of Genetics, National Institute on Aging, US National Institutes of Health, Baltimore, MD USA; 19 Medical Research Council Biostatistics Unit, University of Cambridge, Cambridge, UK; 20 Department of Endocrinology, Clinic of Medicine, St. Olavs hospital Trondheim University Hospital, Trondheim, Norway; 21 HUNT Research Centre, Department of Public Health and Nursing, NTNU – Norwegian University of Science and Technology, Levanger, Norway; 22 Department of Epidemiology Research, Statens Serum Institute, Copenhagen, Denmark; 23 Department of Cardiology, Copenhagen University Hospital, Copenhagen, Denmark; 24 Department of Human Genetics, University of Michigan, Ann Arbor, MI USA; 25 Clinic of Medicine, St. Olavs hospital Trondheim University Hospital, Trondheim, Norway

**Keywords:** Biomarkers, Genome-wide association studies

## Abstract

Iron is essential for many biological processes, but iron levels must be tightly regulated to avoid harmful effects of both iron deficiency and overload. Here, we perform genome-wide association studies on four iron-related biomarkers (serum iron, serum ferritin, transferrin saturation, total iron-binding capacity) in the Trøndelag Health Study (HUNT), the Michigan Genomics Initiative (MGI), and the SardiNIA study, followed by their meta-analysis with publicly available summary statistics, analyzing up to 257,953 individuals. We identify 123 genetic loci associated with iron traits. Among 19 novel protein-altering variants, we observe a rare missense variant (rs367731784) in HUNT, which suggests a role for *DNAJC13* in transferrin recycling. We further validate recently published results using genetic risk scores for each biomarker in HUNT (6% variance in serum iron explained) and present linear and non-linear Mendelian randomization analyses of the traits on all-cause mortality. We find evidence of a harmful effect of increased serum iron and transferrin saturation in linear analyses that estimate population-averaged effects. However, there was weak evidence of a protective effect of increasing serum iron at the very low end of its distribution. Our findings contribute to our understanding of the genes affecting iron status and its consequences on human health.

## Abstract (web summary)

A GWAS on the four major iron-related biomarkers is conducted using data from the Trøndelag Health Study (HUNT), Michigan Genomics Initiative (MGI), and SardiNIA study, to identify 123 loci associated with iron homeostasis and effect on all-cause mortality.

## Introduction

Iron is essential for a variety of physiological processes in the human body, but excess iron is toxic. Iron overload is associated with a wide range of health problems, including liver damage, type 2 diabetes, cardiovascular disease, and neurodegenerative diseases such as Alzheimer’s disease^1–3^, while long-term iron deficiency causes anemia, which can disrupt cognitive function and the immune system^4–6^. Because of the damaging effects of both deficiency and overload, iron metabolism is tightly regulated^7^.

Iron is bound, transported, and delivered around the body by the transferrin glycoprotein^8^, while the main intracellular iron storage, ferritin, provides a long-term reserve of iron for the formation of hemoglobin and other heme proteins^9–11^. Serum iron, serum ferritin, transferrin saturation percentage (TSP), and the total iron-binding capacity (TIBC) of transferrin are biochemical measurements that are commonly used together to assess an individual’s iron status^12^. Both high and low TSP have been associated with an increased mortality risk in observational studies^13–16^, although the underlying pathophysiologic mechanisms are unclear.

Mutations in various iron metabolism genes can cause both iron deficiency and overload^17–19^. Genetic variants in the transferrin gene, *TF*, and in the homeostatic iron regulator gene, *HFE*, have been estimated to account for about 40% of genetic variation in transferrin levels^20^. A recent genome-wide association study (GWAS) meta-analysis^21^ of serum iron, ferritin, TSP, and TIBC from Iceland, UK, and Denmark reported 46 novel loci associated with at least one of these biomarkers, implicating proteins involved in iron homeostasis. Identifying additional genetic loci associated with iron status could further increase our understanding of pathophysiologic mechanisms underlying dysregulated iron levels. Furthermore, genetic variants from the most recent study^21^ could improve existing genetic risk scores (GRS) that have been widely used to assess the causal associations of iron status on a range of outcomes using Mendelian Randomization^22–27^ (MR). However, the new GRSs have not yet been validated in an independent study. Further, despite the observed damaging effects of both very high and very low iron stores, no previous MR studies have investigated the shape of the associations between genetically proxied iron status biomarkers and mortality. By validating the most recent genetic risk scores and using MR in an independent study (HUNT), we provide robust and novel insights into the causal associations between iron status biomarkers and all-cause mortality, particularly regarding non-linear relationships.

Here, we identify 123 loci associated with iron status, by combining three approaches: (i) genome-wide association studies of variants deeply imputed from the TOPMed reference panel^28^ in the Trøndelag Health Study (HUNT)^29^ and the Michigan Genomics Initiative (MGI), as well as variants imputed from a cohort-specific reference panel in SardiNIA^30^ (ii) association tests with genotyped coding variants selected from low-coverage (5×) whole-genome sequencing in HUNT, (iii) genome-wide meta-analyses of HUNT, MGI, SardiNIA and summary statistics from deCODE, Interval and the Danish Blood Donor Study (DBDS)^21^. The analyses included up to 257,953 individuals (57% females, 43% males) with measured iron status biomarkers. We evaluate the variance explained by previously published variants for serum iron, serum ferritin, TSP and TIBC in HUNT. Furthermore, we use the genetic variants for the iron status biomarkers to estimate the average causal effect of a population shift in the biomarker distributions on all-cause mortality (the population-averaged effect), and investigate the shape of the causal relationships using non-linear MR. We find evidence of an average harmful effect of increasing serum iron and transferrin saturation in the general population, but also weak evidence of a protective effect of increasing serum iron at the very low end of its distribution.

## Results

### Discovery of genetic loci associated with iron status

We identified 123 genetic loci associated (*p*-value <5 × 10^−8^) with at least one iron related biomarker (Tables 1–2, Supplementary Data 1, Supplementary Figs. 1–4, and Supplementary Note 1) in genome-wide association meta-analyses of the iron status biomarkers in six cohorts: HUNT, MGI, SardiNIA, deCODE, Interval and DBDS (Supplementary Data 2). To the best of our knowledge, 66 of these loci were novel for any iron status biomarker, while 57 had been reported for at least one of the biomarkers in previous studies. Additionally, among the 57 known iron status loci, 12 loci were for the first time associated with one or more additional biomarkers, further emphasizing the role of these loci in iron-related biological processes. Among the 62 unique index (lowest *p*-value) variants in novel iron status loci that had been imputed in more than one study, 49 had consistent directions of effects across all the analyzed studies. We also identified three novel missense variants associated with at least one biomarker among the variants ascertained from low-pass whole genome sequencing and selected for targeted genotyping in HUNT (Supplementary Data 3).

**Table 1:** Novel loci associated with the iron status biomarkers serum iron, TSP, and TIBC in a genome-wide association meta-analysis.

Trait	rsID-Alt	Effect	SE	*P*-value	MAF	Nearest gene(s)	Consequence	*N*	Studies
TIBC	rs514595-C	0.03	0.005	6.5E−10	0.155	*AKR1A1;NASP*		208,422	H;M;S;dC;I
TIBC S-Iron	rs137891701-D rs61804206-G	0.04 0.03	0.006 0.005	4.9E−9 4.7E−10	0.129 0.142	*SDHC;FCGR2A* *RPL3P1P11;FCRLA*	*R*^2^ = 1.00 w/missense	202,496 236,612	H;M;dC;I H;M;S;dC;I
TIBC	rs1435167-A	−0.02	0.004	1.6E−10	0.373	*EGLN1*	*R*^2^ = 0.89 w/missense	208,422	H;M;S;dC;I
S-Iron	rs7580634-T	−0.02	0.003	4.7E−10	0.464	*IL1F10;IL1RN*		236,612	H;M;S;dC;I
TIBC	rs11900910-T	0.02	0.004	2.1E−11	0.451	*EPAS1*		208,422	H;M;S;dC;I
TIBC	rs1861408-T	0.02	0.004	7.8E−10	0.297	*SERTAD2;LINC01800*		208,422	H;M;S;dC;I
TIBC	rs715-C	0.02	0.004	4.5E−10	0.285	*CPS1*	*R*^2^ = 0.92 w/missense	202,496	H;M;S;dC;I
TIBC	rs781168893-T	−0.75	0.064	2.1E−31	0.003	*SLC12A8;ZNF148*		56,667	H
S-Iron	rs571559677-C	−0.35	0.063	3.1E−8	0.002	*CLSTN2*		67,171	H;M
TIBC	rs760897340-G	−0.97	0.104	7.5E−21	0.001	*DIPK2A;LNCSRLR*		56,667	H
TIBC	rs969672878-A	−0.90	0.103	2.3E−18	0.001	*LOC440982;LINC02032*		56,667	H
TSP	rs218264-T	0.02	0.004	4.2E−8	0.250	*LINC02283;LINC02260*		198,516	H;M;dC;I
TIBC	rs185240714-A	−0.07	0.009	2.4E−13	0.040	*PELO*		204,433	H;M;dC;I
TIBC	rs41274050-T	0.13	0.022	4.8E−9	0.008	*A1CF*		202,496	H;M;dC;I
S-Iron TIBC	rs10740134-C rs10740134-C	0.02 0.02	0.003 0.004	7.8E−11 8.9E−10	0.487 0.487	*REEP3* *REEP3*	*R*^2^ = 0.96 w/missense *R*^2^ = 0.96 w/missense	236,612 198,516	H;M;S;dC;I H;M;dC;I
S-Iron	rs59213222-D	−0.03	0.005	9.3E−9	0.223	*ZMIZ1*		230,682	H;M;S;dC;I
TIBC	rs143878994-A	0.05	0.009	3.6E−8	0.046	*ARHGAP20*		202,496	H;M;S;dC;I
TIBC	rs76895963-G	−0.08	0.015	4.2E−8	0.017	*CCND2-AS1*		202,496	H;M;S;dC;I
TIBC	rs3782735-A	0.02	0.004	2.4E−9	0.402	*LAG3*		202,496	H;M;S;dC;I
S-Iron TSP	rs2900478-A rs2900478-A	−0.02 −0.03	0.004 0.005	3.9E−8 1.4E−10	0.178 0.179	*SLCO1B1* *SLCO1B1*	*R*^2^ = 0.90 w/missense *R*^2^ = 0.90 w/missense	236,612 198,516	H;M;S;dC;I H;M;dC;I
TIBC	rs1150975-A	0.02	0.004	4.1E−8	0.347	*LINC02422;RESF1*		202,496	H;M;S;dC;I
TIBC	rs77371258-A	0.04	0.006	3.5E−13	0.099	*POP5;CABP1*	*R*^2^ = 0.88 w/missense	202,496	H;M;S;dC;I
S-Iron	rs4759827-T	−0.02	0.004	1.5E−8	0.279	*ADGRD1*		236,612	H;M;S;dC;I
S-Iron TSP	rs1958078-C rs1958078-C	0.04 0.04	0.005 0.005	1.0E−14 1.7E−11	0.135 0.131	*SMOC1* *SMOC1*		236,612 198,516	H;M;S;dC;I H;M;dC;I
TIBC	rs12879801-G	0.03	0.004	1.5E−17	0.452	*ADAM21P1;SYNJ2BP-COX16*		202,496	H;M;S;dC;I
TIBC	rs11159099-G	−0.03	0.004	5.2E−16	0.482	*YLPM1*		202,496	H;M;S;dC;I
TIBC	rs8062982-C	0.03	0.004	6.7E−12	0.401	*CHD9;LOC643802*		202,496	H;M;S;dC;I
TIBC	rs34682685-A	0.04	0.006	1.2E−12	0.100	*HPR*		202,496	H;M;S;dC;I
TIBC	rs2287322-G	0.03	0.004	8.9E−13	0.239	*WDR81*	*R*^2^ = 0.97 w/missense	202,496	H;M;S;dC;I
TIBC	rs1292072-G	0.03	0.004	5.0E−11	0.224	*VMP1;TUBD1*		202,496	H;M;S;dC;I
TIBC	rs34284056-A	−0.02	0.004	1.6E−8	0.264	*ZCCHC2*		202,496	H;M;S;dC;I
TIBC	rs111234557-G	−0.03	0.006	4.0E−9	0.121	*MAU2*		202,496	H;M;S;dC;I
TIBC	rs12976652-C	−0.05	0.006	6.5E−18	0.105	*MYPOP*		202,496	H;M;S;dC;I
S-Iron TIBC	rs1800961-T rs1800961-T	0.04 0.05	0.008 0.009	2.4E−8 4.0E−9	0.047 0.047	*HNF4A* *HNF4A*	Missense Missense	236,612 202,496	H;M;S;dC;I H;M;S;dC;I
S-Iron TSP	rs3747207-A rs3747207-A	0.03 0.02	0.004 0.004	1.7E−12 1.1E−8	0.234 0.233	*PNPLA3* *PNPLA3*	*R*^2^ = 1.00 w/missense *R*^2^ = 1.00 w/missense	236,612 198,516	H;M;S;dC,I H;M;dC;I

Associated traits: Serum iron (S-iron), Transferrin Saturation Percentage (TSP), Total Iron Binding Capacity (TIBC). Locus index variant with alternate allele/indel (rsID-Alt, D = deletion) per associated trait. Effect size and direction (Effect) with standard error (SE) and functional consequence (correlation *R*^2^ reported if index variant is strongly correlated (*R*^2^ > 0.8) with a missense variant.) is given for the alternate variant. Minor allele frequency (MAF), sample size (N), studies: HUNT (H), MGI (M), deCODE (dC), Interval (I), SardiNIA (S), DBDS (D). See Supplementary Data 1 for further details.

**Table 2:** Novel loci associated with the iron status biomarker ferritin in a genome-wide association meta-analysis.

rsID-Alt	Effect	SE	*P*-value	MAF	Nearest gene(s)	Consequence	*N*	Studies
rs477190-A	−0.02	0.003	1.3E−08	0.404	*FAM43B;CDA*		257,953	H;M;dC;I;D
rs604126-G	0.02	0.004	2.7E−08	0.314	*BCL2L11;MIR4435-2HG*		257,953	H;M;dC;I;D
rs17050272-A	0.02	0.003	1.1E−09	0.417	*LINC01101;GLI2*		257,953	H;M;dC;I;D
rs17707216-A	0.04	0.007	2.2E−08	0.076	*ZSWIM2;CALCRL*		257,953	H;M;dC;I;D
rs553656123-A	0.11	0.020	1.1E−08	0.008	*NBEAL2*		257,953	H;M;dC;I;D
rs142350264-I	−0.04	0.007	1.7E−09	0.098	*BSN*		246,139	dC;I;D
rs6822746-A	−0.02	0.004	6.3E−09	0.317	*SRD5A3-AS1;TMEM165*		257,953	H;M;dC;I;D
rs4865796-A	0.02	0.004	6.4E−09	0.319	*ARL15*		257,953	H;M;dC;I;D
rs79694859-A	0.05	0.009	3.2E−09	0.046	*PDE4D*		257,953	H;M;dC;I;D
rs35107257-A	0.04	0.008	1.1E−08	0.058	*OCLN*		257,953	H;M;dC;I;D
rs970079-G	−0.02	0.003	7.0E−09	0.463	*RGMB*		257,953	H;M;dC;I;D
rs35486885-G	−0.06	0.008	5.2E−12	0.038	*HLA-DQB2;HLA-DOB*		257,953	H;M;dC;I;D
rs13215052-A	−0.03	0.005	5.7E−09	0.111	*TFEB*		257,953	H;M;dC;I;D
rs189899297-A	0.02	0.004	4.7E−08	0.263	*ATP6V0D2;SLC7A13*		246,139	dC;I;D
rs681099-C	−0.02	0.003	1.7E−08	0.455	*EIF3E*		255,619	M;dC;I;D
rs72775768-T	−0.02	0.004	4.2E−09	0.259	*INPP5E*		257,953	H;M;dC;I;D
rs9423600-G	0.02	0.004	4.8E−08	0.268	*LINC02561;UCN3*		257,953	H;M;dC;I;D
rs704017-G	−0.02	0.003	2.6E-08	0.378	*ZMIZ1-AS1*		257,953	H;M;dC;I;D
rs17112021-G	0.02	0.004	2.0E−10	0.275	*NKX2-3;SLC25A28*		257,953	H;M;dC;I;D
rs7102016-T	0.02	0.003	3.4E−08	0.444	*SBF2*		257,953	H;M;dC;I;D
rs10750215-T	0.02	0.004	1.5E-08	0.417	*MIR100HG;UBASH3B*		257,953	H;M;dC;I;D
rs9512463-C	−0.02	0.004	4.8E−09	0.226	*GPR12;USP12*		257,953	H;M;dC;I;D
rs28715334-T	0.02	0.004	6.4E−09	0.203	*SLC7A8*		257,953	H;M;dC;I;D
rs764195359-G	−0.30	0.051	2.6E−09	0.002	*MGA*		255,619	M;dC;I;D
rs11634990-C	−0.03	0.005	2.0E−09	0.170	*IREB2*		257,953	H;M;dC;I;D
rs62074125-C	0.02	0.004	3.8E−08	0.288	*WNT3*		257,953	H;M;dC;I;D
rs2952290-A	−0.02	0.004	1.9E−08	0.214	*PRKAR1A*	*R*^2^ = 0.84 w/missense	257,953	H;M;dC;I;D
rs141253118-A	−0.12	0.023	4.6E−08	0.005	*NOTCH3;EPHX3*		255,619	M;dC;I;D
rs2595586-G	−0.02	0.003	2.2E−08	0.414	*ATRN*		257,953	H;M;dC;I;D
rs6088374-C	−0.04	0.005	1.9E−16	0.169	*LINC00028;HM13*		255,619	M;dC;I;D
rs540828131-G	−0.16	0.026	1.3E−09	0.007	*HMOX1*		255,619	M;dC;I;D

Locus index variants with alternate allele/indel (rsID-Alt, I = insertion). Effect size and direction (Effect) with standard error (SE) and functional consequence (correlation *R*^2^ reported if index variant is strongly correlated (*R*^2^ > 0.8) with a missense variant) given for the alternate variant. Minor allele frequency (MAF), sample size (N), studies: HUNT (H), MGI (M), deCODE (dC), Interval (I), SardiNIA (S), DBDS (D). See Supplementary Data 1 for further details.

Genes in several associated loci coded for proteins with established functions in iron homeostasis (*TF* [transferrin]) *SLC25A37* [mitoferrin-1], *SLC25A28* [mitoferrin-2], *SLC11A2* [divalent metal-transporter 1] and *SLC40A1* [ferroportin-1], *HFE* [homeostatic iron regulator], *TFRC* [transferrin receptor], *TFR2* [transferrin receptor 2], *HAMP* [hepcidin], *ERFE* [erythroferrone], *HMOX1* [heme oxygenase], *IREB2* [iron responsive element binding protein 2], *EPAS1* [endothelial PAS Domain Protein 1] and *TMPRSS6* [transmembrane serine protease 6])^7,31–33^ Four of the loci that had not been reported for iron status biomarkers in previous GWAS studies, contained known iron related genes (*SLC25A28*, *HMOX1*, *IREB2,* and *EPAS1*), providing additional confidence in the reported associations. With exception of *HAMP* and *TFR2*, these genes were the nearest gene to the index variant in the locus.

### Protein-altering variants in meta-analysis loci

We identified 32 protein-altering single nucleotide polymorphisms (SNPs) in the meta-analysis, which were either index variants or variants in strong linkage disequilibrium (LD) (*R*^2^ > 0.8 or *D*’ = 1.0) with an index variant (Supplementary Data 1). In addition to SNPs known to be related to diseases such as hemochromatosis, atransferrinemia or iron deficiency anemia^19,34–38^, and variants that had previously been reported for at least one of the iron traits^21,39^, we identified 11 protein-altering variants in novel iron status loci (Supplementary Data 4): rs9427398 (*FCGR2A*), rs2437150 (*SPRTN*), rs1047891 (*CPS1*), rs41274050 (*A1CF*), rs1935 (*JMJD1C*), rs3742049 (*COQ5*), rs4149056 (*SLCO1B1*), rs2070863 (*SERPINF2*), rs883541 (*WIPI1*), rs1800961 (*HNF4A*) and rs738409 (*PNPLA3*). In known iron status loci, we further identified eight protein-altering variants not previously reported for any of the analyzed iron traits: rs367731784 (*DNAJC13*), rs3812594 (*SEC16A*), rs34376913 (*C9orf163*), rs445520 (*SLC11A2*), rs28929474 (*SERPINA1*), rs737700 (*C16orf71*), rs77542162 (*ABCA6*) and rs34654230 (*RCN3*).

### Custom genotyped variants in HUNT: Protein-altering variants in iron status loci

Among the targeted candidate variants in HUNT identified by sequencing and clinical studies, we identified three additional, novel protein-altering variants (Supplementary Data 3) that were not included in the meta-analyses, and which were associated with iron status biomarkers. These were located in *NRM* (rs374815811), *HLA*-*DRB5* (rs701884), and *TFR2* (chr7:100629337:A:T, GRCh38).

### Heritability and genetic correlation of iron status markers

We estimated the respective narrow-sense SNP heritability (variance explained, *V*_*g*_/*V*_*p*_ ± 1 standard error) of serum iron (0.15 ± 0.01), TIBC (0.43 ± 0.01) and TSP (0.21 ± 0.01) in HUNT using Genome-wide Complex Trait Analysis (GCTA)^40^. We found pair-wise genetic correlations between 11% and 75% (Supplementary Data 5) for the four iron status biomarkers using LD Score Regression^41^ with the meta-analysis summary statistics. The TSP phenotype was derived from the serum iron and TIBC measurements, giving rise to the two strongest genetic correlations. The weakest correlation (iron vs TIBC) did not reach nominal significance (*p*-value = 0.35).

### Functional mapping

We used Bayesian colocalization analysis to identify 86 unique pairs of GWAS loci and cis-expression quantitative trait locus (cis-eQTL) signals that showed sufficient overlap in at least one tissue to be consistent with a shared causal variant for the gene expression and the iron status biomarker (Supplementary Data 6). We found associations in a range of tissues which highlighted genes with established roles in iron metabolism (*TF* [posterior probability of a common causal variant, PP4 = 0.96], *TMPRSS6* [PP4 = 0.82], *ERFE* [PP4 = 0.97–0.98], *IREB2* [PP4 = 0.80]*, SLC40A1* [PP4 = 0.79–0.96])^17,31^. Additionally, our results confirmed previously reported genes (*DUOX2* [PP4 = 0.76], *HBS1L* [PP4 = 0.98], *IL6R* [PP4 = 0.81–0.82], *SLC25A37* [PP4 = 0.85], *ABO* [PP4 = 0.97], *RNF43* [PP4 = 0.99])^21,39^, and identified novel genes interacting with previously reported genes, for example *DUOXA2*^42^. Several iron status loci were also colocalized with cis-eQTL signals for genes in the major histocompatibility complex (MHC) other than *HFE*^43^, as well as with transcription regulators^44–46^, additional transporter proteins^47,48^ and transferases^49,50^.

Using Data-driven Expression Prioritized Integration for Complex Traits (DEPICT)^51^ we found an enriched (false discovery rate [FDR] < 0.05) expression of ferritin associated genes in the urogenital system, digestive system, and the hemic and immune system (Supplementary Data 7). Serum iron, TSP, and TIBC associated genes were not enriched in any tissue types at FDR < 0.05, however, the strongest enrichment for genes in all three traits were found in liver tissue, and particularly in hepatocytes (TSP, TIBC). The top ten genes per trait when prioritized based on similarity between the associated (*p*-value < 5 × 10^−8^) loci, included known iron regulatory genes (*TFR2*, *HAMP*, *TFRC*, and *SLC40A1*), genes in which we had identified protein-altering variants (*IL6R*, *F5*, *GCKR*, *DUOX2*, *SERPINA1*, *ABCA6*, and *SLCO1B1*), genes we found in the colocalization analysis (*DUOXA2*, *IL1RN*, and *SLC25A37*), as well as genes predicted to have iron ion binding and heme binding properties in gene ontology analyses (*CYP3A43*, *CYP3A5*)^52^ (Supplementary Data 8). Finally, we used DEPICT and found gene sets enriched with iron status associated genes (Supplementary Data 9). All the top ten gene sets enriched with iron associated loci and seven with TSP associated loci reached FDR < 0.05: One iron associated gene set, and five TSP associated gene sets were related to the liver (including abnormal liver physiology and gene sets related to metabolic processes), but also to inflammation (acute-phase response, decreased leukocyte cell number), coagulation (coagulation factor protein-protein interaction networks) and neurodevelopment (abnormal myelination). The top ten gene sets enriched with ferritin and TIBC associated loci included decreased circulating iron levels, decreased spleen iron levels, gene sets related to red blood cells (decreased hemoglobin, decreased hematocrit, erythrocyte homeostasis, and differentiation), as well as liver fibrosis and liver inflammation. However, these gene sets did not reach FDR < 0.05. We identified the 1% top-ranked genes per trait based on both physical distance to the associated genetic variants and functional similarity to other associated genes (Supplementary Data 10–13) using Polygenic Priority Scores (PoPS)^53^. The prioritized genes included the main known iron regulatory genes, several genes that were nearest to the meta-analysis index variants, and novel genes in which we identified protein-altering variants (*WIPI1*, *SERPINF2*, and *HNF4A*), further supporting a role for these genes in iron biology.

### Phenome-wide association study (PheWAS) of biomarker loci

**Fig. 1:**
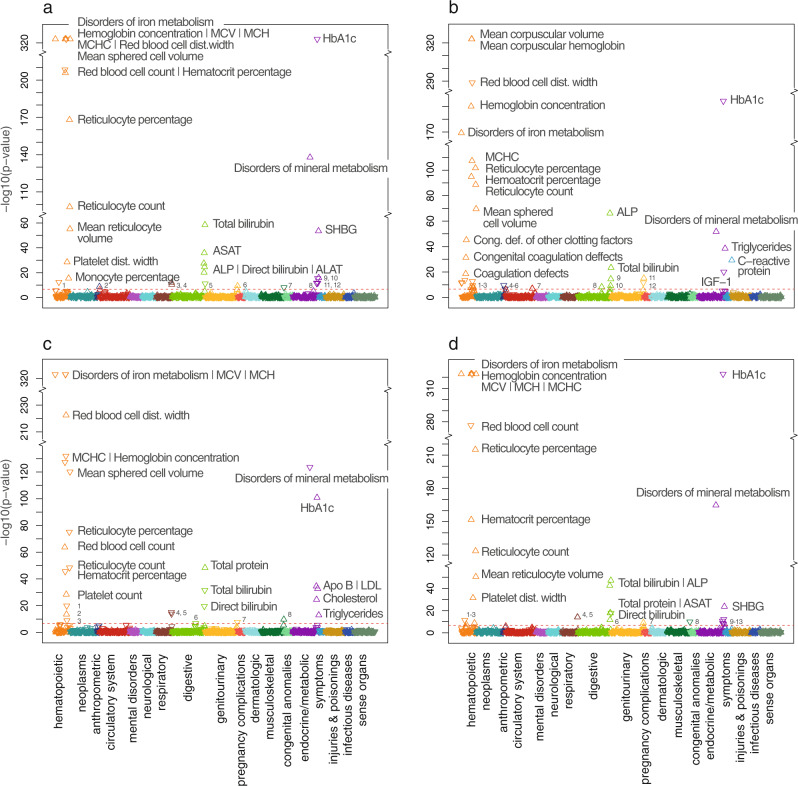
Phenome-wide associations between GRSs for iron status biomarkers and 1473 phecodes, blood biomarkers, and continuous traits in the UK Biobank. Phenome-wide associations between the GRS for each biomarker (serum iron [**a**], serum ferritin [**b**], total iron-binding capacity [**c**], and transferrin saturation percentage [**d**]) and 1473 phecodes, blood biomarkers, and continuous traits in the UK Biobank. Triangles pointing upwards indicate a positive association between the phenotype and the GRS (where a higher GRS score represents higher level of the biomarker) and vice versa. Associations with *p*-values < 10^−324^ are plotted at 10^−324^. The Bonferroni corrected *p*-value cut-off (2.3 × 10^−7^) is given as a red dotted line. Biological domains are indicated by color; for the significant associations, these are: hematopoietic (orange), anthropometric (dark blue), circulatory system (red), respiratory (brown), digestive (light green), genitourinary (yellow), musculoskeletal (dark green), endocrine/metabolic (magenta), symptoms (light blue). Abbreviations: MCV mean corpuscular volume, MCH mean corpuscular hemoglobin, MCHC mean corpuscular hemoglobin concentration, dist. distribution, Cong. def. congenital deficiencies, HbA1c glycated hemoglobin, SHBG sex hormone-binding globulin, LDL low-density lipoproteins, Apo B apolipoprotein B, IGF-1 insulin-like growth factor 1, FEV1 forced expiratory volume (1 s), FVC forced vital capacity, ALP alkaline phosphatase, ASAT aspartate aminotransferase, ALAT alanine aminotransferase, GGT gamma-glutamyl transferase. **a**: ^1^Other anemias, ^2^Impedance, ^3^FEV1, ^4^FVC, ^5^Total protein, ^6^Cystatin C, ^7^Phosphate, ^8^Apo B, ^9^Testosterone, ^10^IGF-1, ^11^Cholesterol, ^12^LDL. **b**: ^1^Anemias (iron deficiency and other anemias), ^2^Platelet indices (platelet count, platelet crit, platelet dist. width), ^3^White blood cell counts (lymphocytes, leukocytes, monocytes), ^4^Seated height, ^5^Water mass, ^6^Fat-free mass, ^7^Phlebitis and thrombophlebitis, ^8^Non-alcoholic liver disease, ^9^Gamma-glutamyl Transferase, ^10^Direct bilirubin, ^11^Urate, ^12^Creatinine. **c**: ^1^Mean platelet volume, ^2^Platelet crit, ^3^Platelet dist. width., ^4^FEV1, ^5^FVC, ^6^Liver cirrhosis, ^7^Cystatin C, ^8^Phosphate. **d**: ^1^Other anemias, ^2^Monocyte percentage, ^3^Congenital deficiency of other clotting factors, ^4^FEV1, ^5^FVC, ^6^ALAT, ^7^Cystatin C, ^8^Phosphate, ^9^LDL, ^10^Apo B, ^11^IGF-1, ^12^Cholesterol, ^13^Testosterone.

In total, 105 of the meta-analysis index variants were significantly associated (*p*-value < 2.3 × 10^−7^) with at least one additional disease phenotype (‘phecode’^54^), blood biomarker, or continuous trait in the UK Biobank. Of the 1473 phenotypes tested, 129 were significantly associated with at least one variant (Supplementary Data 14–19). The associations spanned numerous biological domains, but most associations were within the hematopoietic (615 variant-trait associations), endocrine/metabolic (303 variant-trait associations), and digestive (243 variant-trait associations) domains. The strongest (*p*-values < 1 × 10^−300^) and most numerous associations were seen for red blood cell-related traits (hemoglobin concentration, mean corpuscular hemoglobin, mean corpuscular volume, red blood cell distribution width, red blood cell count, hematocrit percentage, mean reticulocyte volume), where we found associations with variants in *HFE* (rs1800562, rs144861591, and rs79220007), *TMPRSS6* (rs855791, rs2076085), *HBS1L*;*MYB* (rs9399136, rs56293029), *LINC002283*;*LINC002260* (rs218264), *HK1* (rs17476364) and *IKZF1* (rs12718598). The strongest associations also included associations between ‘disorders of iron metabolism’ and the *HFE* variants (rs1800562, rs144861591 and rs79220007), platelet related traits (platelet count, platelet crit, mean platelet volume) and variants in *HBS1L*;*MYB* (rs9399136, rs56293029) and *REEP3* (rs10740134), direct and total bilirubin and the *SLCO1B1* variant rs2900478, alkaline phosphatase and rs10740134 (*REEP3*), rs186021206 (*ASGR2*;*ASGR1*) and rs9987289 (*LOC157273*), C-reactive protein and rs2228145 (*IL6R*) and rs35945185 (*LEPR*;*PDE4B*), sex hormone-binding globulin (SHBG) and rs10740134 (*REEP3*), glycated hemoglobin (HbA1c) and rs17476364 (*HK1*), and triglycerides and rs1260326 (*GCKR*), rs112875651, rs28601761 and rs2954027 (*TRIB1*;*LINC0086*). Overall, all GRSs for the four iron status biomarkers were associated with disorders of mineral metabolism, in particular iron metabolism, as well as with red blood cell-related traits and other blood composition measures, lipid-related measurements, direct and total bilirubin, and HbA1c (Fig. 1 and Supplementary Data 17–19). Several GRSs were associated with anemias (iron, ferritin, TSP) and coagulation defects (ferritin, TSP), the GRS for TIBC was associated with liver cirrhosis without mention of alcohol, and the GRS for ferritin was associated with chronic non-alcoholic liver disease, and with phlebitis and thrombophlebitis. Finally, some of the GRSs were also associated with other continuous traits and biomarkers, including markers related to liver damage (iron, TSP, ferritin) and SHBG (iron, TSP), which is associated with liver iron overload^55^.

### Linear Mendelian randomization

The linear MR (ratio of coefficients method) indicated an increased mortality risk with increased serum iron and TSP, with the point estimates suggesting that an increase of 1 standard deviation (SD) in both serum iron (1 SD = 6.3 µmol L^−1^) and TSP (1 SD = 11.3 percentage points) would lead to an increased risk of mortality of 7% (Table 3). The estimates for ferritin and TIBC were not statistically significant, however the point estimates for a 1 standard deviation increase in serum ferritin (1 SD = 46 µg L^−1^) and TIBC (1 SD = 9.2 µmol L^−1^) were a 7% increase and 4% decrease in mortality, respectively (Table 1). The estimate for ferritin was also very imprecise.

**Table 3:** Linear Mendelian randomization ratio of coefficient estimates.

Biomarker	*N*	Hazard ratio (95% CI)	*P*-value
Serum iron	56,654	1.07 (1.01–1.14)	0.03
Serum ferritin*	2335	1.07 (0.26–4.41)	0.92
TSP	56,651	1.07 (1.02–1.12)	0.01
TIBC	56,654	0.96 (0.92–1.01)	0.10

Hazard ratios with 95% confidence intervals for all-cause mortality are given per 1 standard deviation increase in the biomarker. Sample size (N), Confidence interval (CI), Transferrin saturation percentage (TSP), Total iron-binding capacity (TIBC).

*Measured in fertile, non-pregnant women, 20–55 years old, with no blood donations in the two previous years.

### Non-linear Mendelian randomization

**Fig. 2:**
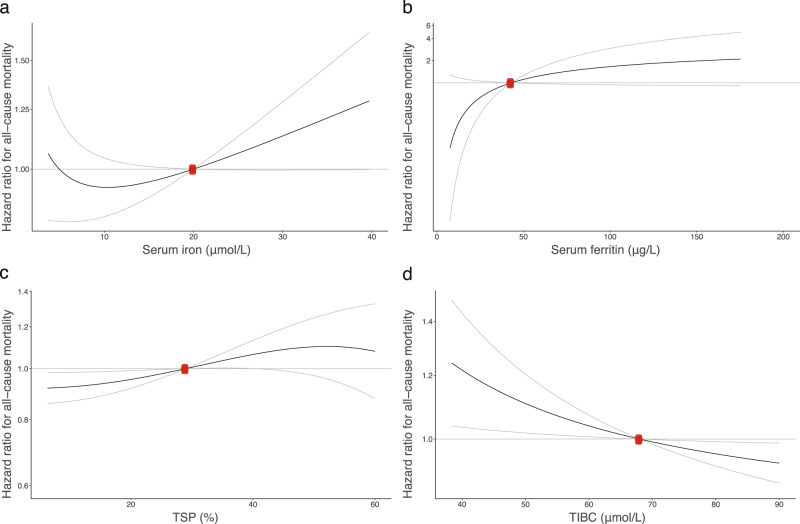
Non-linear Mendelian Randomization: causal associations between iron status biomarkers and all-cause mortality. Dose-response curves (black) between iron traits and all-cause mortality in HUNT (gray lines give 95% confidence interval). The *x*-axis gives **a**: serum iron levels (µmol/L) [*N* = 56,654], **b**: serum ferritin (µg L^−1^) [*N* = 2335], **c**: transferrin saturation (%) [*N* = 56,651] and **d**: total iron-binding capacity (TIBC) (µmol L^−1^) [*N* = 56,654]. The *y*-axis gives the hazard ratios for all-cause mortality with respect to the reference values (red dot), which represent the established target values (iron, TIBC, TSP)^84^ or median value (ferritin) for the traits. The curve gradients represent the localized average causal effect at each point. *N* = sample size.

To investigate a potential non-linear causal association between iron status and all-cause mortality, we used GRSs as instruments for serum iron (F-statistic = 3,618, *R*^2^ = 0.06), TIBC (F-statistic = 8,373, *R*^2^ = 0.129), TSP (F-statistic = 6811, *R*^2^ = 0.107) and ferritin (F-statistic = 37.81, *R*^2^ = 0.015) in a non-linear MR analysis and estimated the shape of the associations between the genetically predicted traits and all-cause mortality (Fig. 2 and Supplementary Data 20–23). The median follow-up time was 23.6 years. After performing a statistical test for whether the best-fitting non-linear model of degree 1 fitted the data better than a linear model (*p*-values: 0.50, 0.09, 0.24, and 1 for iron, ferritin, TIBC, and TSP respectively), we generally did not find strong statistical evidence supporting a non-linear relationship over a linear one for the associations between any of the genetically proxied iron traits and all-cause mortality. However, the point estimates for serum iron did follow a J-shape, with a negative slope at very low levels of serum iron and a constant positive slope above 10 µmol L^−1^. The point estimates were however imprecise at the tails of the distribution. The other analyses indicated a lower risk at higher TIBC and lower TSP and ferritin levels, with a weak indication (*p*-value = 0.09) of a non-linear effect for ferritin. Post hoc sensitivity analyses using genetic instruments that were consistent with systemic iron status (increased iron, ferritin, and TSP, and decreased TIBC) rather than just representing a single biomarker, gave similar results (Supplementary Fig. 5).

## Discussion

We performed the largest GWAS meta-analysis to-date of iron status biomarkers and identified 123 genetic loci (78 novel for at least one biomarker) associated with iron status biomarkers, including 19 novel protein-altering variants. Although 78 loci were classified as novel for at least one of the tested biomarkers, many of them had known associations with the other tested biomarkers. Further, a high number of the loci were associated with red blood cell indices both in the current and previous studies^56,57^, thereby strengthening our confidence in the role of these loci in iron homeostasis. The strong associations with red blood cell indices are in line with the findings from previous studies^21,39^. Because iron traits are biologically linked, we might expect to find the same loci associated with several of the tested traits, as seen for loci with established roles in iron homeostasis (e.g., *HFE, TF,* and *TMPRSS6*). We confirmed the genetic similarity between iron status biomarkers by observing a high genetic correlation of 75% between serum iron and TSP, which is expected for dependent variables. However, most of the loci for TIBC and ferritin were specific for those traits, which was reflected by lower genetic correlations between some of the measured biomarkers.

Overall, our findings are consistent with established knowledge about iron homeostasis and the role of iron in various biological processes: In four of the novel loci for iron status biomarkers, the genes nearest to the index variant encoded proteins with established roles in iron regulation: (1) the mitochondrial iron transporter mitoferrin-2 (*SLC25A28*), (2) heme oxygenase 1 (*HMOX1*), which catalyzes heme degradation, (3) the iron-responsive element binding 2 (*IREB2*), and (4) *PAS1*, which regulates erythropoiesis according to cellular iron availability^32^. In addition to the direct associations with iron-related traits, many of the genes in both the known and novel loci were known for their role in other biological processes and diseases, such as cardiovascular or liver markers, immunity, inflammation, and cancers, suggesting that these loci might be more indirectly associated with iron status, either via processes that cause or are caused by changes in iron status. Knowing that ferritin and transferrin are also acute-phase biomarkers^58^, such indirect associations might also explain the high number of TIBC and ferritin-associated loci that were not associated with other iron status biomarkers. The PheWAS analyses further linked the identified loci to many different traits and phenotypes, particularly within the hematopoietic, digestive, and endocrine/metabolic domains.

The novel protein-altering variants were also found in genes associated with diverse biological traits and functions, which potentially highlight the many biological processes both involved in and dependent on iron and iron regulation. These included, but were not limited to, genes involved in or associated with: (1) Iron gut absorption, regulation, and transport (*TFR2, SLC11A2,* and *DNAJC13*)^8,54,59,60^, where we found a rare (minor allele frequency (MAF) = 0.0009) protein-altering variant with moderate effect size in *DNAJC13*, a gene suggested to be involved in transferrin recycling^59^. This variant was only imputed in HUNT, where it was more than 100 times more common than in other non-Finnish Europeans (https://gnomad.broadinstitute.org/variant/rs367731784)^61^; (2) Parkinson’s disease, which is associated with iron deposition in the brain^62^, and where *DNAJC13* is one of several associated genes whose roles in the disease are still debated^63^; (3) Concentrations of hemoglobin (*SPRTN*, *FCGR2A*, *CPS1, PNPLA3,* and *ABCA6*)^53,64–66^, which holds more than two thirds of the body’s iron^1^, and bilirubin (*SLCO1B1*)^67^, which together with iron are products of heme degradation; (4) Iron-dependent (putative) tumor suppression (*JMJD1C*)^68^; (5) Fibrinolysis and bleeding (*SERPINF2*)^69^; (6) Liver-related traits (*ABCA6*, *HNF4A, PNPLA3,* and *SERPINA1*)^69–71^; and (7) Tissue specific iron accumulation (*WIP1* could potentially be associated with iron accumulation in the brain via its homolog *WIPI4*^72^, and the index variant we report in *SERPINA1*, rs28929474, has recently been proposed as a modifier of HFE-related hereditary hemochromatosis^73^ and as a trigger for hepatic iron overload^74^); A previous study also identified a different protein-altering SNP in the serine protease inhibitor *SERPINA1*^21^. The transmembrane serine protease 6 (encoded by *TMPRSS6*) is a negative regulator of hepcidin^75^, a key hormone regulator of iron homeostasis^17^. Given the role of the transmembrane serine protease 6 in iron regulation, both serine protease inhibitors could potentially affect iron regulation via this gene. In contrast to some of the well-known genetic variants associated with iron homeostasis, most of the novel protein-altering variants in the current study were predicted as “tolerated” by in silico testing. This is however as expected for common variants, since an increased GWAS sample size will give an increased power to detect variants with smaller effect sizes than what has been identified in previous studies.

Using DEPICT, we detected an enriched expression in the liver of genes in iron status loci. This is in line with the important role of the liver in iron metabolism and storage^76^, including hepcidin production. Consistent with previous studies, our analysis prioritized genes encoding known hepcidin regulators such as *HFE*, *TMPRSS6*, *TF*, *TFRC*, *TFR2*, *ERFE*, and *IL6R*^77–80^. Mutations in several of these genes have been demonstrated to cause diseases of iron deficiency or overload^1,19,37,81^. The associations with other genes related to inflammation, both in the DEPICT and colocalization analyses, could possibly be related to the hepcidin response to inflammation. The genes and gene sets prioritized by DEPICT pointed to several different biological processes, which might reflect the numerous roles of iron in the body. A limitation with using a similarity-based method for gene set and gene prioritization for iron traits was that the software excluded the MHC region from the analysis, thereby also excluding one of the most central genes in iron homeostasis, the *HFE* gene.

Colocalization analysis further linked the GWAS loci both to the liver and to iron overload: iron status loci overlapped with cis-eQTLs for several of the hepcidin regulators, and genes involved in other liver functions such as lipid and fatty acid metabolism (*ORMDL1, FADS1*)^82,83^. The latter was also found in a previous independent GWAS study^39^ and is in line with the results from the PheWAS analysis. The colocalization of iron status loci and cis-eQTLs were however found in several tissues, and not primarily in liver. A limitation to the analysis was however due to different sample sizes for each tissue, where liver had a relatively small sample size and subsequently lower power than other tissues. Further, gene expression is highly dependent on the cellular conditions, so tissue from living donors might not produce similar outcomes.

Because iron plays an essential role in so many biological processes, several MR studies have explored the causal effect of iron status on a range of diseases^22–27^. Despite the known harmful effects of both iron deficiency and overload, as well as the associations of both high and low TSP with increased mortality risk in traditional observational studies^13–16^, no previous MR studies have investigated the shape of the exposure-outcome relationship. We, therefore, assessed the causal effect of iron status biomarkers on all-cause mortality and investigated the shape of these associations. We demonstrated that the GRSs based on the previous study were good instruments for iron, TSP, TIBC, and ferritin in the independent HUNT study (variance explained 6% (iron), 11% (TSP), 13% (TIBC), 1.6% (ferritin)), thereby validating the previous study findings. Using these, we found evidence of a harmful effect of increased serum iron and TSP (derived from serum iron and TIBC) in linear analyses that estimated population-averaged effects. The point estimates of TIBC and ferritin were also suggestive of a harmful effect of increased iron status, although the estimates were not statistically significant, and the ferritin estimate was very imprecise due to the small sample size. In non-linear models, we did not find strong statistical evidence supporting non-linear relationships over linear ones. However, there was weak evidence of a protective effect of increasing serum iron at the very low end of its distribution, at serum iron levels below the normal range of 10–34 µmol L^−1^ ^84^. The results were supported by post-hoc sensitivity analyses using only genetic variants consistent with systemic iron status. Our findings confirm the previously reported associations between elevated TSP and an increased risk for overall mortality. We also found weak support for the previously reported J-shaped association between iron biomarkers and mortality, although further studies are needed to confirm this.

This study had several clear limitations. First, in our GWAS analyses we did not adjust for additional factors that could affect the biomarker concentrations, such as iron supplementation, inflammatory status, alcohol consumption, and menopausal status (except for ferritin, where the full sample was pre-menopausal). These factors could therefore have influenced the effect estimates, particularly for rare variants. Second, we would need a larger sample size to confirm a non-linear shape of the exposure-outcome relationship at the extremes of the biomarker distributions. The analysis of ferritin was particularly limited by the small sample (*N* = 2334) consisting of only relatively young, non-pregnant females, giving a low number of strata and (fortunately) few deaths. Third, the association of the GRSs with all-cause mortality could be attenuated because HUNT participants with suspected iron deficiency anemia or phenotypic hemochromatosis were later contacted by the primary health care services and offered treatment, and they could therefore have obtained a healthier iron status than they would otherwise have had, causing the analysis to be less precise. Finally, although the four biomarkers are commonly used to assess people’s iron status, neither of them is a very good individual predictor of iron stores, and the findings should therefore be interpreted with caution.

In summary, we have increased the number of iron status-associated loci through a large GWAS meta-analysis and validated the latest genetic risk scores for four iron status biomarkers. We find evidence of a harmful population-averaged effect of genetically proxied elevated serum iron and TSP, and weak evidence of a protective effect of increasing serum iron in individuals at the very low end of its distribution. Our findings contribute to our understanding of the genes affecting iron status and its consequences on human health.

## Methods

### Cohort descriptions

#### HUNT

The HUNT Study is a longitudinal population-based health study conducted in the county of Trøndelag, Norway since 1984^29^. About 123,000 individuals (aged ≥ 20 years) have participated in at least one of four surveys, and more than 70,000 of these participants have been genotyped using one of three Illumina HumanCoreExome arrays: 12 v.1.0, 12 v.1.1 and 24 with custom content (UM HUNT Biobank v1.0). Samples whose genotypes had call rates < 99%, estimated contamination > 2.5%, large chromosomal copy number variants, lower call rate of a technical duplicate pair or twin, gonosomal constellations other than XX or XY, or discrepancy between inferred sex and reported gender were excluded. Following genotyping, variants with call rate < 99%, deviations from Hardy Weinberg equilibrium (*p*-value < 10^−4^ in unrelated samples of European ancestry), probe sequences that could not be perfectly mapped to the reference genome, cluster separation < 0.3, Gentrain score < 0.15, or if another assay with higher call rate had genotyped the same variant were excluded. All variants were imputed from the TOPMed reference panel (freeze 5)^28^ using Minimac4 v1.0 (https://genome.sph.umich.edu/wiki/Minimac4). The reference panel is composed of 53,831 multi-ethnic samples and 410,323,831 SNP and indel variants at high depth (mean read depth 38.2X). Variants with a minor allele count (MAC) > 10 or imputation *R*^2^ ≥ 0.3 were included in analysis. A subset of individuals was genotyped with additional custom content variants.

#### MGI

The MGI is a repository of genetic data and electronic medical records from Michigan Medicine. Approximately 80,000 participants (aged ≥18 years) have predominantly been enrolled prior to surgical procedures with over 59,000 individuals genotyped using Illumina Infinium CoreExome-24. Following genotyping, sample-level QC was performed to remove sex-mismatches, duplicates, samples with call rate < 99%, or with estimated contamination > 2.5%. Variants with GenTrain score < 0.15, Cluster Separation scores < 0.3, Hardy–Weinberg Equilibrium *p*-value among unrelated individuals of European ancestry <1 × 10^−4^, or with evidence of batch effects (*p*-value < 1 × 10^−3^, Fisher’s exact test) were excluded. Imputation was performed using the TOPMed Imputation Server (v1.2.7). Variants with MAF > 0.05% and imputation quality *R*^2^ ≥ 0.3 were included in analysis.

#### SardiNIA

The SardiNIA study is a longitudinal population-based health study including 6,602 individuals from the Lanusei valley on Sardinia. The participants have been genotyped on four different Illumina Infinium arrays, OmniExpress, Cardio-Metabochip^85^, Immunochip^86^, and Exome Chip). Samples with low call rate or with discrepancies between inferred and reported sex and/or relationships were excluded. After genotyping, variants with low call rates, large discordance among duplicate or identical twin genotypes, excess Mendelian inconsistencies, deviations from Hardy–Weinberg equilibrium, or MAF = 0 were excluded. Variants were then imputed from a SardiNIA-specific sequencing panel (~4× coverage) of 3839 individuals, using Minimac3^87^. Markers with imputation quality *R*^2^ > 0.3 (or >0.6 in variants with MAF < 1%) were retained, resulting in a total of ~19 million genetic variants.

### Iron status biomarkers

Distributions of the biomarker levels in the HUNT, MGI, and SardiNIA participants included in the current study are reported in Supplementary Data 2.

#### HUNT

Non-fasting serum samples were drawn in 1995–1997 (HUNT2). Serum iron concentration (µmol L^−1^) was determined using a FerroZine method using a Hitachi 911 Autoanalyzer (reagents from Boehringer, Germany). The serum transferrin concentration (µmol L^−1^) was analyzed by an immunoturbidimetric method using the Hitachi 911 Autoanalyzer (reagents from DAKO A/S, Denmark), and calculated for a molecular weight of 79,570 Da. TIBC was calculated as 2 × the serum transferrin concentration. The TSP was calculated as 100 × [serum iron concentration/TIBC]. Serum ferritin (µg L^−1^) was measured from serum samples using an Abbott AxSYM analyzer (reagents from Abbott Laboratories, USA). In total, 56,667 HUNT participants had measurements of serum iron and TIBC, 56,664 had measurements of TSP, while ferritin was only measured in 2334 women (fertile, non-pregnant, aged 20–55 years).

#### SardiNIA

Serum iron (µmol L^−1^) and serum transferrin concentrations (µmol L^−1^) were measured in fasting blood-samples from individuals with genotype and imputation data from the SardiNIA cohort. TIBC was calculated as 2 × the serum transferrin concentration. In total, 5930 and 5926 genotyped SardiNIA participants had measurements on serum iron and TIBC respectively.

#### MGI

Serum iron concentration (µmol L^−1^) was measured using the Ferrozine Colorimetric assay, and serum ferritin (µg L^−1^) was measured using a Chemiluminescent Immunoassay. Serum transferrin concentrations were measured using an Immunoturbidimetric assay, and TIBC was calculated as 2 × the serum transferrin concentration. The TSP was calculated as 100 × [serum iron concentration/TIBC]. For individuals with multiple measurements, the initial measurement was used in the analyses. In total, 10,403, 9480, 10,399, and 10,381 participants from MGI had measurements of serum iron, serum ferritin, TIBC, and TSP respectively.

### Association analyses

Association analyses of all iron traits (iron, ferritin, TIBC, and TSP) in HUNT were performed using a linear mixed model regression under an additive genetic model for each variant as implemented in BOLT-LMM v2.3.4^88^, which also controls for relatedness between the samples. Association analyses of all iron traits in MGI were performed using a linear regression model in unrelated individuals using rvtests^89^. In both HUNT and MGI, we applied rank-based inverse normal transformation on the iron variables after adjusting for age and sex using linear regression, and included age, sex, genotyping batch, and the first 10 principal components (PCs) of ancestry as covariates. Association analyses of serum iron and TIBC in SardiNIA were performed using age, age^2^, and sex-adjusted inverse-normalized residuals of TIBC or iron as input to the Efficient Mixed Model Association eXpedited (EMMAX)^90^ single variant test as implemented in EPACTS (https://github.com/statgen/EPACTS).

Additionally, we performed association analyses of serum iron, TIBC, and TSP in HUNT with 19,273 additional polymorphic custom content variants genotyped in 44,248 (serum iron, TIBC) or 44,246 individuals (TSP) using BOLT-LMM v2.3.4^88^, including the same covariates and rank-based normal transformation of the variables as was done in the main analyses.

### Meta-analyses

We performed fixed-effect inverse-variance weighted meta-analysis of summary statistics for iron (sample size *N* = 236,612), ferritin (*N* = 257,953), TIBC (*N* = 208,422) and TSP (*N* = 198,516) using METAL^91^. Serum iron and TIBC were meta-analyzed in all studies (HUNT, MGI, SardiNIA, and summary statistics from deCODE and Interval). SardiNIA did not have data on serum ferritin and TSP and was therefore excluded from these meta-analyses, while the available summary statistics for ferritin also included the DBDS study. To harmonize genomic positions from each study, we used LiftOver from UCSC^92^ to map the data from SardiNIA from Human Genome Build GRCh37 to GRCh38. Because standard errors were not given in the available summary statistics from deCODE, Interval and DBDS, we calculated them as the absolute value of the (effect size/qnorm(*p*-value/2)), where qnorm represents the inverse standard normal distribution. Prior to meta-analysis, we filtered all studies on MAF ≥ 0.001, and in HUNT, MGI and SardiNIA we performed genomic control correction of any analyses with an inflation factor λ > 1. We considered genetic loci reaching a *p*-value < 5 × 10^−8^ for follow-up analyses.

### Definition of independent loci and locus novelty

Genetic loci were defined around variants with a genome-wide significant association with a trait (*p*-value < 5 × 10^−8^). The locus borders were set 500 kb to each side of the highest and lowest genetic positions reaching genome-wide significance in each region. Overlapping genetic loci were merged if the index (lowest *p*-value) variants were in LD (*R*^2^ ≥ 0.2 and/or *D*’ ≥ 0.8), or if one of the index variants was too rare to calculate LD with the other variants from our reference panel of 5000 unrelated individuals in HUNT. A locus was classified as novel for a given trait if it had not been reported previously for the trait, and novel for iron status biomarkers if it had not been previously reported for any of the four traits. Previously published variants were identified through a literature search and a look-up in the GWAS catalog^93^.

### Annotation of genetic variants

We used plink v1.9^94^ with a reference panel of 5000 unrelated individuals in HUNT to identify genetic variants in strong LD (*R*^2^ ≥ 0.8) with the index variants, and annotated the functional consequences and rsIDs of the index variants and LD proxies using ANNOVAR (v.2019Oct24)^95^ and the UCSC human genome browser^92^.

### Functional mapping of genetic variants

We used three different bioinformatic approaches to perform functional mapping and gene prioritization of the meta-analysis summary level data: Bayesian colocalization analysis^96,97^, DEPICT^51^, and Polygenic Priority Scores^98^.

To assess if any iron status loci were overlapping with statistically significant (*p*-value < 5 × 10^−8^) cis-eQTL signals and consistent with shared causal variants for iron status markers and gene expression levels in specific tissue types, we used Bayesian colocalization analysis (‘coloc’) as implemented in the R package coloc. We used cis-eQTL data from 27 general tissue types (49 subtypes) in the individuals of European ancestry from the Genotype-Tissue Expression (GTEx) portal, data set v8 (https://www.gtexportal.org), and the GWAS meta-analysis results for each of the iron status biomarkers as input. For each tissue type, we analyzed all genes whose expression were associated (*p*-value < 5 × 10^−8^) with at least one iron status associated variant (*p*-value < 5 × 10^−8^), using effect sizes and standard errors for each variant-trait association as input. The coloc software estimated the variance in each trait (iron trait or gene expression level) from the sample sizes and minor allele frequencies. We set the prior probability of a genetic variant being associated with only iron traits, only gene expression, or both traits to be 10^−4^, 10^−4^, and 10^−6^ respectively. We considered posterior probabilities (PP4) above 75% to give support for a common causal variant for the iron trait and expression of the gene in the given tissue.

We performed gene set enrichment, gene prioritization, and tissue/cell type enrichment tests on the iron trait loci (*p*-value < 5 × 10^−8^) using DEPICT (v1.1, release 194)^51^. Prior to the analysis, we used LiftOver from the UCSC^92^ to convert the genomic positions of the genetic variants from GRCh38 to GRCh37. Enrichment results with an FDR < 5% were considered significant.

Finally, we prioritized genes by computing Polygenic Priority Scores^98^ from summary-level data from each iron status biomarker. The method uses Multi-marker Analysis of GenoMic Annotation (MAGMA)^99^ to compute gene-level associations and gene-gene correlations from the meta-analysis *p*-values and sample sizes and LD information from individuals of European ancestry from the 1000 Genomes reference panel^100^. MAGMA is applied a second time to perform enrichment analysis for genetic features. Genes are finally prioritized based on a combination of physical distance to associated genetic variants and functional similarity with other associated genes. We considered the 1% top-ranked genes per biomarker to be prioritized genes for the respective traits.

### Heritability estimation

We estimated the narrow-sense additive SNP heritability of serum iron, TIBC, and TSP in HUNT using GCTA^40^. Ferritin heritability was not estimated because of the low sample size in HUNT. We created genetic relationship matrices (GRMs) based on 358,956 genotyped autosomal variants in 56,667 individuals with serum iron and TIBC data, and 56,664 individuals with TSP data. We used the respective GRMs with GCTA-GREML (genomic-relatedness-based restricted maximum-likelihood) to estimate the variance in each variable that was explained by the genetic variants. We used age, sex, and genotyping batch as covariates in the analyses, and we transformed the iron trait variables to normality with rank-based inverse normal transformation after regression on age and sex prior to the analyses.

### Genetic correlation between iron status biomarkers

We used the LD Score Regression software^41^ with the iron status biomarker meta-analysis summary statistics and precomputed LD Scores for Europeans from the 1000 Genomes reference panel^100^, and estimated the pair-wise genetic correlations of the four iron traits. Prior to the analysis, we changed all *p*-values < 1 × 10^−300^ to the exact value 1 × 10^−300^ to make sure the software was able to read the smallest values and did not discard these SNPs. To ensure that only well-imputed SNPs were included in the analysis and thereby avoid bias due to variable imputation quality, we filtered the input files to the HapMap3 reference panel prior to the analysis, as recommended by the software developers (https://github.com/bulik/ldsc/).

### Phenome-wide association tests (PheWAS)

We constructed GRSs for the iron status biomarkers by summing the product of the effect size and the estimated allele count (dosage) for the index variants in genome-wide significant loci (*p*-value < 5 × 10^−8^). We used TOPMed imputed estimated allele counts and effect sizes from the meta-analysis and calculated the GRS for participants of white British ancestry in the UK Biobank. We tested for pleiotropic associations of each GRS (GRS-PheWAS) and individual index variant (single variant PheWAS) with 1 394 phecodes and 79 continuous traits and blood biomarkers. We used a logistic or linear regression model respectively to assess the association of the single variant estimated allele counts (‘dosage’) or inverse normalized GRS and each phecode or continuous trait/biomarker. For the GRS-PheWAS we included as covariates sex, birth year and the first four principal components of ancestry. For the single variant PheWAS we used publicly available GWAS summary statistics (phecodes from https://pheweb.org/UKB-TOPMed/ and continuous traits and biomarkers from https://pan.ukbb.broadinstitute.org/). To correct for multiple testing, we used a Bonferroni corrected *p*-value significance cut-off of 2.3 × 10^−7^, correcting for the number of tested variants, GRSs, phecodes, biomarkers, and continuous traits. Two variants were excluded from the single variant PheWAS and 14 from the GRS-PheWAS because they were not imputed in UK Biobank (Supplementary Note 2).

### Validation of genetic risk scores in HUNT

To validate the previously published results from Iceland, Denmark, and UK, we created weighted GRSs for each trait based on the published index variants and effect sizes^21^ using the same approach as described in the previous section. We tested the predictive ability of each GRS by regressing each trait on the respective GRS in the independent cohort, HUNT (*N*_iron_ = *N*_TIBC_ = 56,667, *N*_TSP_ = 56,664, *N*_ferritin_ = 2334), and report the trait variance explained by the GRS. In total, ten variants were excluded from the GRSs because they were not imputed in HUNT (Supplementary Note 3).

### Mendelian randomization of iron status on all-cause mortality

To assess the causal association of iron status on all-cause mortality, we performed linear MR analyses using the ratio of coefficients method^101^, using GRSs as genetic instruments for the four iron-related traits. The GRSs were constructed as described above, using index variants and external effect sizes from the previous independent meta-analysis^21^. We used linear regression to estimate the associations between the iron-related traits and the GRS, and a Cox proportional hazards regression to estimate the association of the GRS with mortality. The MR estimate was obtained as the ratio of the outcome-instrument and exposure-instrument association estimates. The standard error was estimated as the standard error of the GRS-mortality association divided by the GRS-biomarker association estimate.

To further assess the shape of the association, we performed a non-linear MR with the fractional polynomial method^102,103^ in HUNT, using the same GRS as genetic instrument for the iron traits. The method has been described in detail elsewhere^103–106^. In brief, each iron-related trait was regressed on its respective GRS, including appropriate covariates, and the population was divided into 100 (iron, TIBC, TSP) or 20 (ferritin) strata based on the residual traits. We stratified on the residual traits to avoid overadjustment and collider bias, as the residual trait was defined as the residual from the regression of the biomarker on the GRS, and represented the predicted biomarker level if the GRS had been zero. The number of strata was reduced for ferritin because of the lower sample size for this biomarker. In each stratum, we used linear regression to estimate the association of the GRS with the iron trait, and Cox proportional hazards regression to estimate the association of the GRS with mortality. We calculated the localized average causal effect (LACE) of the respective trait on all-cause mortality in each stratum as the ratio of the GRS-outcome and GRS-exposure associations. Unless the fractional polynomial of degree 1 fit as good (*p*-value > 0.5) as that of degree 2, we plotted the best-fitting fractional polynomials of degree 2 to allow for flexibility in the non-linear biomarker-mortality relationship. Otherwise, we plotted the best-fitting fractional polynomials of degree 1. We performed meta-regression of the LACE against the mean of the exposure in each stratum and tested whether the best-fitting fractional polynomial of degree 1 fitted the LACE estimates better than a linear model using the fractional polynomial method^102^.

To further validate the selection of SNPs representing each biomarker in the non-linear MR, we performed post-hoc sensitivity analyses rerunning the non-linear MR method with new instruments that had stricter criteria for SNP inclusion. Here, we restricted the GRSs to index variants from the previous study^21^ that were not only GWAS significant for at least one trait, but also nominally significant (*p*-value < 0.05) for the remaining traits. Further, we excluded SNPs that had directions of effect that were not consistent with systemic iron status (increasing serum iron, ferritin, and TSP, and decreasing TIBC)^107^. We used the remaining 14 SNPs (Supplementary Note 4) to construct each of the four GRSs in the analysis as described for the main analysis.

### Statistics and reproducibility

Unless otherwise specified, statistical analyses were performed in R v.3.6.4. Sample sizes, biomarker, age, and sex distributions per study included in the GWAS meta-analyses are given in Supplementary Data 2.

### Ethics

All study participants have given informed consent. The analyses in HUNT has approval from the Norwegian Data Protection Authority and the Regional Ethics Committee for Medical and Health Research Ethics in Central Norway (REK Reference Number: 2014/144), the analyses in MGI are approved by the Institutional Review Board of the University of Michigan Medical School (IRB Reference Number: HUM00094409), the analyses in SardiNIA are approved by the local ethics committee for the Istituto di Ricerca Genetica e Biomedica-CNR (IRGB-CNR; Cagliari, Italy), and the analyses in UK Biobank are covered by the ethics approval for UK Biobank studies (application 24460) from the NHS National Research Ethics Service on 17th June 2011 (Ref 11/NW/0382) and extended on 10th May 2016 (Ref 16/NW/0274).

### Reporting summary

Further information on research design is available in the Nature Research Reporting Summary linked to this article.

## Supplementary information

Supplementary Information

Description of Additional Supplementary Files

Supplementary Data 1 - 23

Reporting Summary

## Data Availability

The data supporting the findings are available in the Supplementary Data and upon request. The UK Biobank data can be obtained by application (https://www.ukbiobank.ac.uk). Summary level data from previously published meta-analysis in deCODE, Interval, and DBDS are available from https://www.decode.com/summarydata/. Variant associations with phecodes, continuous traits, and biomarkers used to generate Fig. 1 are accessible from https://pheweb.org/UKB-TOPMed/ and https://pan.ukbb.broadinstitute.org/. The data underlying Fig. 2 are given in Supplementary Data 20–23. The GWAS meta-analysis summary level data from the current study are available from NTNU Open Research Data, https://dataverse.no/dataverse/ntnu (10.18710/S9TJEL).

## Code availability

We used the following publicly available software and data (URLs) to generate and analyze the data: R v.3.6.4, BOLT-LMM v.2.3.4 (https://alkesgroup.broadinstitute.org/BOLT-LMM/downloads), EMMAX (https://github.com/statgen/EPACTS), METAL v.2011-03-25 (https://genome.sph.umich.edu/wiki/METAL), UCSC LiftOver command line tool (http://genome.ucsc.edu/cgi-bin/hgLiftOver), ANNOVAR v.2019Oct24 (https://annovar.openbioinformatics.org), plink v1.9 (https://zzz.bwh.harvard.edu/plink), DEPICT (https://data.broadinstitute.org/mpg/depict), Coloc (https://chr1swallace.github.io/coloc/articles/a01_intro.html), PoPS v0.1 (https://github.com/FinucaneLab/pops), GCTA (https://cnsgenomics.com/software/gcta), LD Score Regression (https://github.com/bulic/ldsc) and SUMMnlmr (https://github.com/amymariemason/SUMnlmr).
