# Supplementary material for: Genome-wide meta-analysis of iron status biomarkers and the effect of iron on all-cause mortality in HUNT
Source: Commun Biol. 2022 Jun 16;5:591. doi: 10.1038/s42003-022-03529-z (PMC9203493; doi:10.1038/s42003-022-03529-z)
Supplement: Supplementary file 1 — Supplementary Information [file 42003_2022_3529_MOESM1_ESM.pdf]

Supplementary information for:

Genome-wide meta-analysis of iron status biomarkers and the effect of iron on all-cause mortality in HUNT

Marta R Moksnes,[1,†] Sarah E Graham,[2] Kuan-Han Wu[3], Ailin Falkmo Hansen,[1] Sarah A Gagliano Taliun,[4,5] Wei Zhou,[6,7] Ketil Thorstensen,[8] Lars G Fritsche,[9,10] Dipender Gill,[11,12,13,14] Amy Mason,[15] Francesco Cucca,[16,17] David Schlessinger,[18] Gonçalo R Abecasis,[10] Stephen Burgess,[15,19] Bjørn Olav Åsvold,[1,20,21] Jonas B Nielsen,[1,2,22,23] Kristian Hveem,[1,21,*] Cristen J Willer,[1,2,5,24,*] Ben M Brumpton.[1,21,25,†*]

1 K.G. Jebsen Center for Genetic Epidemiology, Department of Public Health and Nursing, NTNU – Norwegian University of Science and Technology, Trondheim, Norway
2 Division of Cardiovascular Medicine, Department of Internal Medicine, University of Michigan, Ann Arbor, MI, USA
3 Department of Computational Medicine and Bioinformatics, University of Michigan, Ann Arbor, MI, USA
4 Department of Medicine and Department of Neurosciences, Université de Montréal, Montréal, QC, Canada
5 Montréal Heart Institute, Montréal, QC H1T 1C8, Canada
6 Analytic and Translational Genetics Unit, Department of Medicine, Massachusetts General Hospital, Boston, MA, USA
7 Stanley Center for Psychiatric Research, Broad Institute of MIT and Harvard, Cambridge, MA, USA
8 Department of Clinical Chemistry, St. Olavs hospital Trondheim University Hospital, Trondheim, Norway
9 Department of Biostatistics, University of Michigan School of Public Health, Ann Arbor, MI, USA
10 Center for Statistical Genetics, University of Michigan School of Public Health, Ann Arbor, MI, USA
11 Department of Epidemiology and Biostatistics, School of Public Health, Imperial College London, London, UK
12 Clinical Pharmacology and Therapeutics Section, Institute for Infection and Immunity, St George's, University of London, London, UK
13 Clinical Pharmacology Group, Pharmacy and Medicines Directorate, St George's University Hospitals NHS Foundation Trust, London, UK
14 Novo Nordisk Research Centre Oxford, Old Road Campus, Oxford, UK
15 British Heart Foundation Cardiovascular Epidemiology Unit, Department of Public Health and Primary Care, University of Cambridge, Cambridge, UK
16 Istituto di Ricerca Genetica e Biomedica, Consiglio Nazionale delle Ricerche (CNR), Cagliari, Italy
17 Dipartimento di Scienze Biomediche, Università degli Studi di Sassari, Sassari, Italy
18 Laboratory of Genetics, National Institute on Aging, US National Institutes of Health, Baltimore, MD, USA
19 Medical Research Council Biostatistics Unit, University of Cambridge, Cambridge, UK
20 Department of Endocrinology, Clinic of Medicine, St. Olavs hospital Trondheim University Hospital, Trondheim, Norway
21 HUNT Research Centre, Department of Public Health and Nursing, NTNU – Norwegian University of Science and Technology, Levanger, Norway
22 Department of Epidemiology Research, Statens Serum Institute, Copenhagen, Denmark
23 Department of Cardiology, Copenhagen University Hospital, Copenhagen, Denmark
24 Department of Human Genetics, University of Michigan, Ann Arbor, MI, USA
25 Clinic of Medicine, St. Olavs hospital Trondheim University Hospital, Trondheim, Norway

* These authors jointly supervised this work.

†Corresponding authors:

marta.r.moksnes@ntnu.no

ben.brumpton@ntnu.no

Supplementary Figures

Supplementary Figure 1: Manhattan Plot - Serum Ferritin

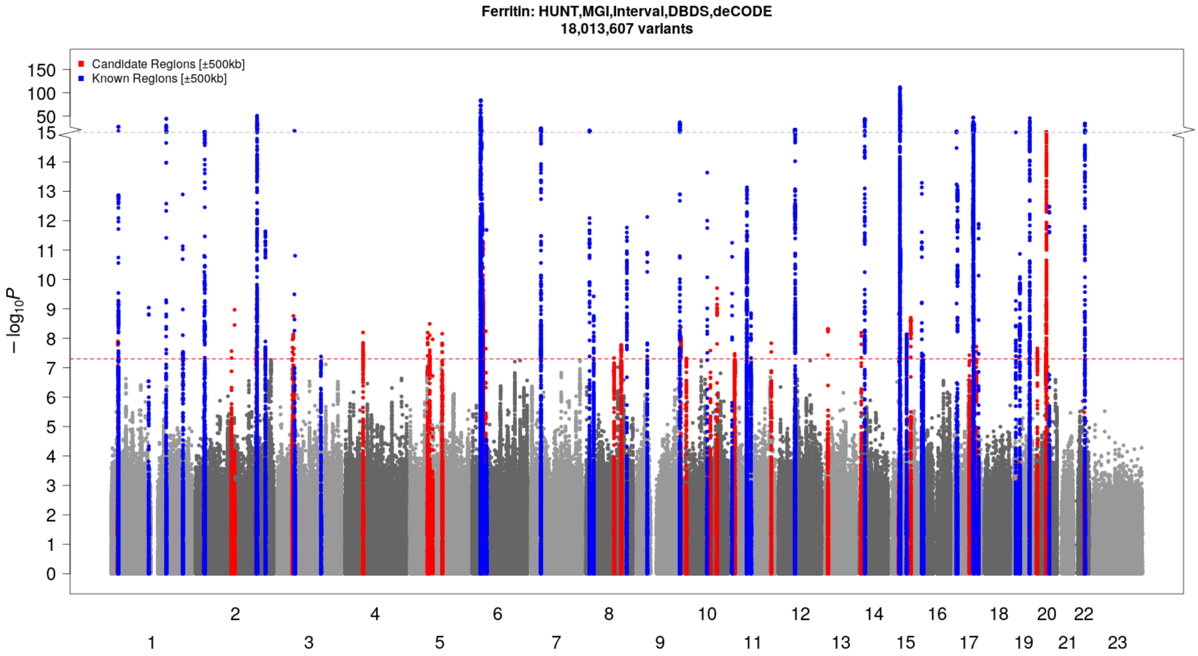

Manhattan plot of the serum ferritin meta-analysis of the HUNT, MGI, deCODE, DBDS and Interval studies: The x-axis gives the chromosomes and chromosomal positions, and the y-axis gives -log10(p-value) for the association of the genetic variants with ferritin. Known (blue) and novel candidate (red) regions for any iron status biomarker.

Supplementary Figure 2: Manhattan Plot - Serum Iron

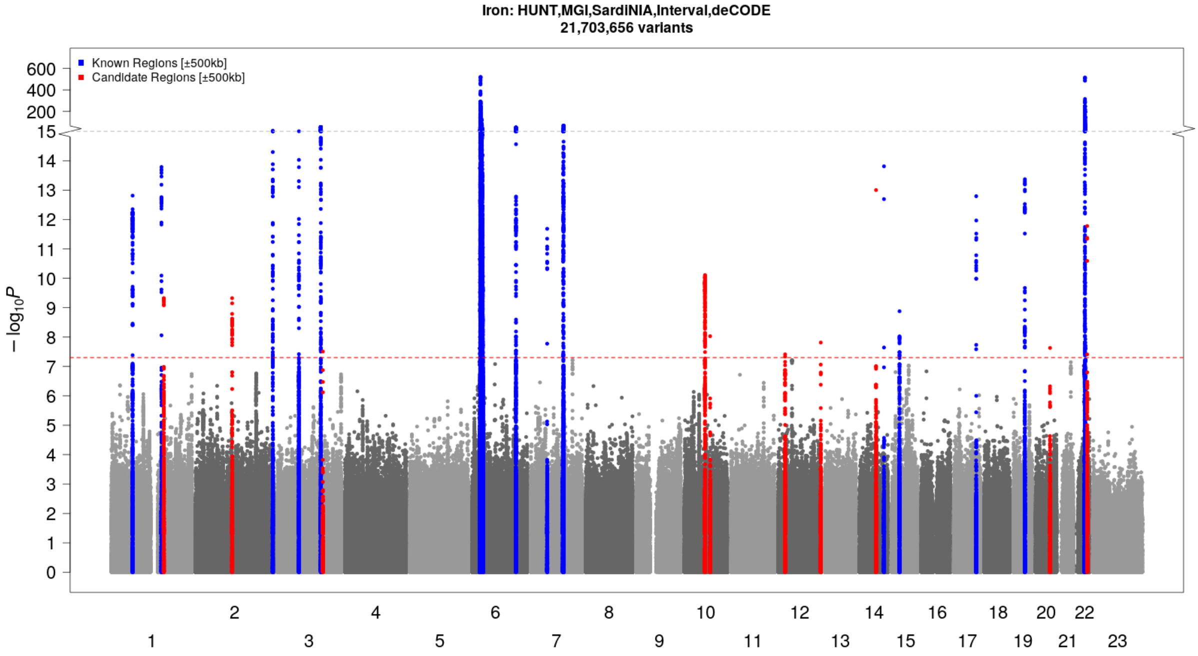

Manhattan plot of the serum iron meta-analysis of the HUNT, MGI, SardiNIA, deCODE and Interval studies: The x-axis gives the chromosomes and chromosomal positions, and the y-axis gives -log10(p-value) for the association of the genetic variants with serum iron. Known (blue) and novel candidate (red) regions for any iron status biomarker.

Supplementary Figure 3: Manhattan plot - Total Iron Binding Capacity

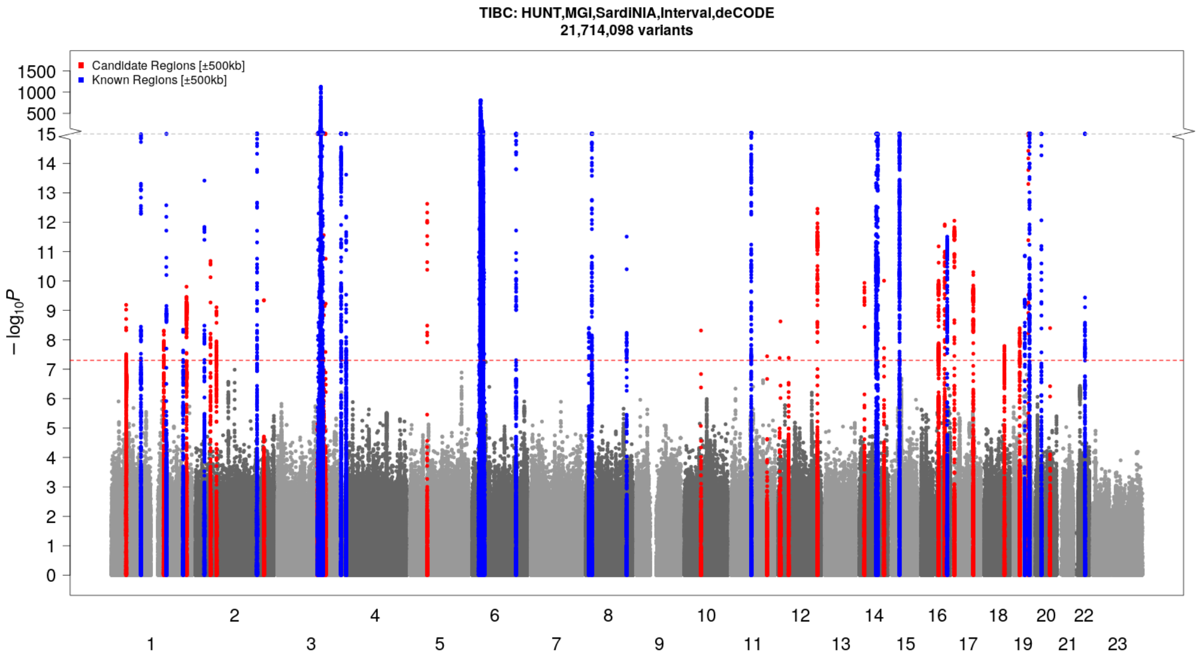

Manhattan plot of the total iron binding capacity (TIBC) meta-analysis of the HUNT, MGI, SardiNIA, deCODE and Interval studies: The x-axis gives the chromosomes and chromosomal positions, and the y-axis gives -log10(p-value) for the association of the genetic variants with TIBC. Known (blue) and novel candidate (red) regions for any iron status biomarker.

Supplementary Figure 4: Manhattan plot - Transferrin Saturation Percentage

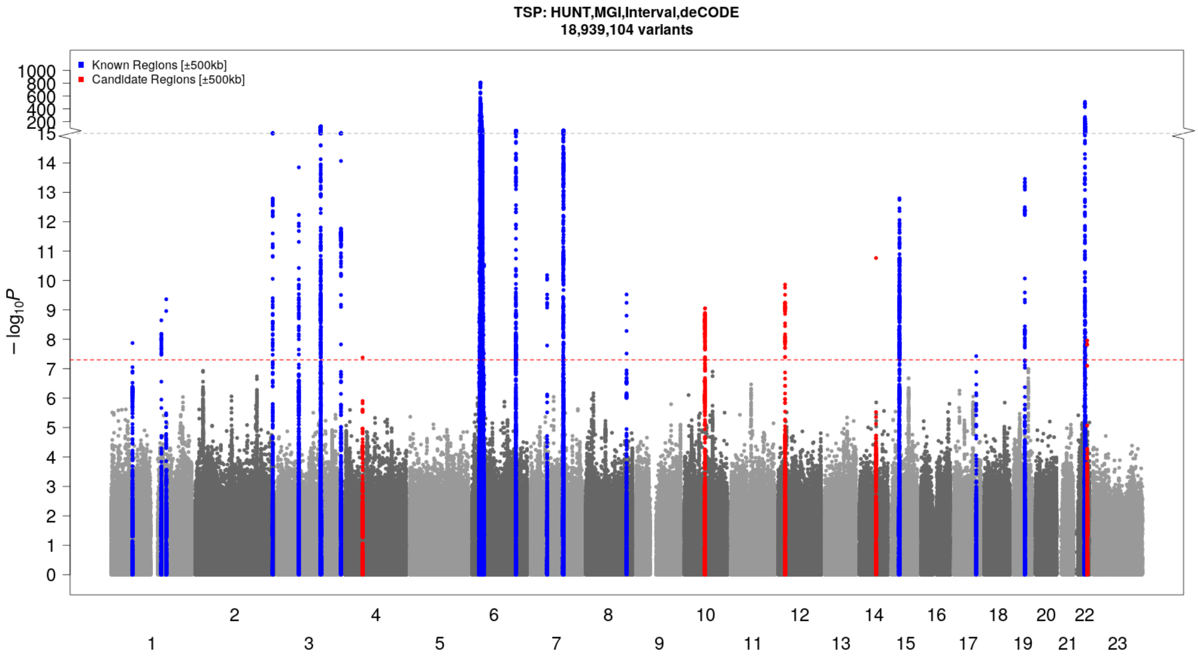

Manhattan plot of the transferrin saturation percentage (TSP) meta-analysis of the HUNT, MGI, deCODE and Interval studies: The x-axis gives the chromosomes and chromosomal positions, and the y-axis gives -log10(p-value) for the associations of the genetic variants with TSP. Known (blue) and novel candidate (red) regions for any iron status biomarkers.

Supplementary Figure 5: Non-linear Mendelian randomization post-hoc sensitivity analysis

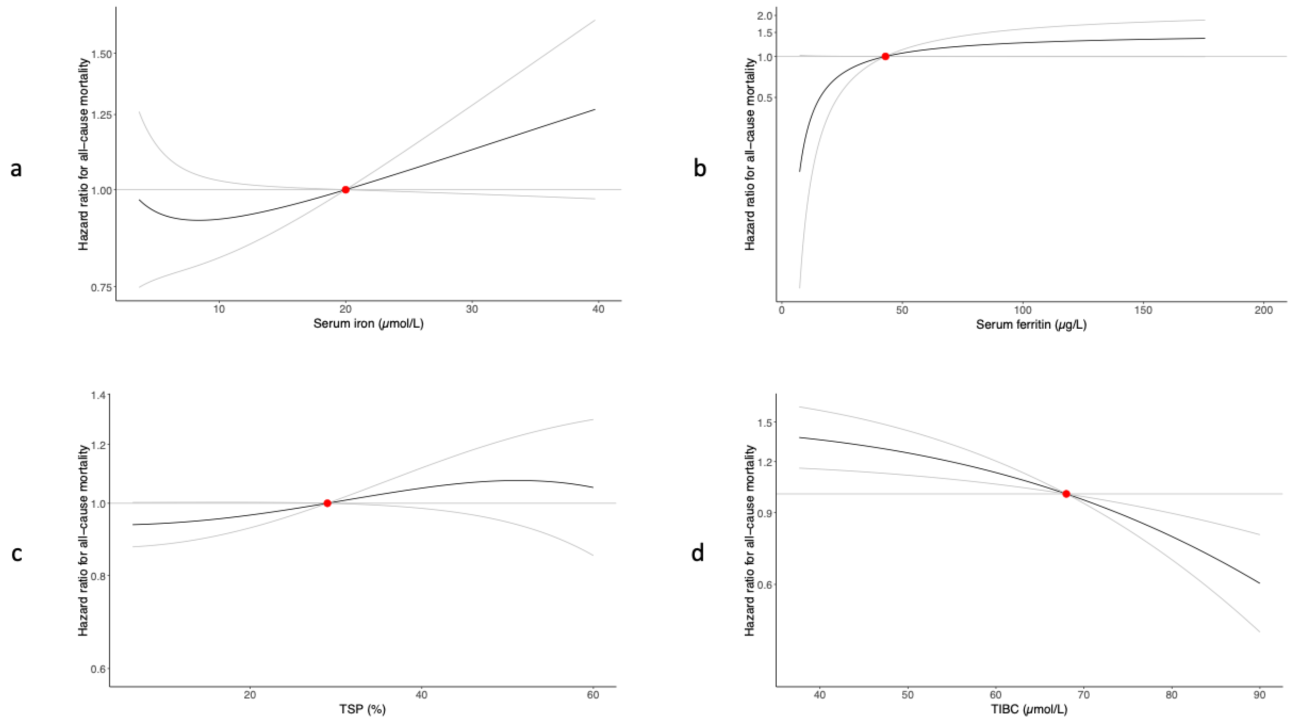

Dose-response curves (black) between iron traits and all-cause mortality in HUNT (gray lines give 95% confidence interval) using a GRS consistent with systemic iron status. The x-axis gives a: serum iron levels (μmol/L), b: serum ferritin (μg/L), c: transferrin saturation (%) and d: total iron binding capacity (TIBC) (μmol/L). The y-axis gives the hazard ratios for all-cause mortality with respect to the reference values (red dot), which represent the established target values (iron, TIBC, TSP)[51] or median value (ferritin) for the traits. The curve gradients represent the localized average causal effect at each point.

Supplementary Notes

Supplementary Note 1: Some novel intronic and intergenic associations reached p-value < 5×10^{-8} in the meta-analysis of deCODE, Interval and DBDS[21], but they were not detected because the previous study design used a consequence specific p-value cut-off prioritizing high-impact variants.

Supplementary Note 2: Genetic variants excluded from GRS-PheWAS because they were not imputed in the UK Biobank reference data: rs374974760 (iron), rs35945185, rs7165401 (TSP), rs551459670, rs142350264, rs536826368, rs7009973, rs189899297, rs681099, 9:133264504:G:GAAACTGCC, rs10685744, rs141253118, rs192331981, rs6088374 (ferritin). Genetic variants excluded from single variant PheWAS because they were not imputed in the UK Biobank reference data: rs142350264, 9:133264504:G:GAAACTGCC (ferritin).

Supplementary Note 3: Genetic variants excluded from the GRSs validation because they were not imputed in HUNT: rs35945185 (iron), rs748587164 (iron, TSP, TIBC), rs773570300 (iron, TSP), rs551459670 (ferritin), rs762752083 (ferritin), rs750717575 (ferritin), rs745795585 (ferritin), rs143041401 (ferritin) and two deletions on chromosomes 9 and 12 (ferritin).

Supplementary Note 4: Genetic variants selected for GRS used in post-hoc sensitivity non-linear Mendelian randomization analyses: rs75965181, rs10801913, rs6025, rs13007705, rs1799945, rs1800562, rs9399136, rs4841429, rs13253974, rs2954029, rs57659670, rs34523089, rs2005682, rs855791. The directions of effect for these genetic variants were consistent with systemic iron status (increasing iron, TSP, ferritin, decreasing TIBC). Further, they were GWAS significant for at least one iron related biomarker and at least nominally significant for the other three biomarkers.
