# Supplementary material for: Genome-wide meta-analysis of iron status biomarkers and the effect of iron on all-cause mortality in HUNT
Source: Commun Biol. 2022 Jun 16;5:591. doi: 10.1038/s42003-022-03529-z (PMC9203493; doi:10.1038/s42003-022-03529-z)
Supplement: Supplementary file 2 — Description of Additional Supplementary Files [file 42003_2022_3529_MOESM2_ESM.pdf]

Description of Additional Supplementary Files for:

Genome-wide meta-analysis of iron status biomarkers and the effect of iron on all-cause mortality in HUNT

Marta R Moksnes,[1,†] Sarah E Graham,[2] Kuan-Han Wu[3], Ailin Falkmo Hansen,[1] Sarah A Gagliano Taliun,[4,5] Wei Zhou,[6,7] Ketil Thorstensen,[8] Lars G Fritsche,[9,10] Dipender Gill,[11,12,13,14] Amy Mason,[15] Francesco Cucca,[16,17] David Schlessinger,[18] Gonçalo R Abecasis,[10] Stephen Burgess,[15,19] Bjørn Olav Åsvold,[1,20,21] Jonas B Nielsen,[1,2,22,23] Kristian Hveem,[1,21,*] Cristen J Willer,[1,2,5,24,*] Ben M Brumpton.[1,21,25,†*]

1 K.G. Jebsen Center for Genetic Epidemiology, Department of Public Health and Nursing, NTNU – Norwegian University of Science and Technology, Trondheim, Norway
2 Division of Cardiovascular Medicine, Department of Internal Medicine, University of Michigan, Ann Arbor, MI, USA
3 Department of Computational Medicine and Bioinformatics, University of Michigan, Ann Arbor, MI, USA
4 Department of Medicine and Department of Neurosciences, Université de Montréal, Montréal, QC, Canada
5 Montréal Heart Institute, Montréal, QC H1T 1C8, Canada
6 Analytic and Translational Genetics Unit, Department of Medicine, Massachusetts General Hospital, Boston, MA, USA
7 Stanley Center for Psychiatric Research, Broad Institute of MIT and Harvard, Cambridge, MA, USA
8 Department of Clinical Chemistry, St. Olavs hospital Trondheim University Hospital, Trondheim, Norway
9 Department of Biostatistics, University of Michigan School of Public Health, Ann Arbor, MI, USA
10 Center for Statistical Genetics, University of Michigan School of Public Health, Ann Arbor, MI, USA
11 Department of Epidemiology and Biostatistics, School of Public Health, Imperial College London, London, UK
12 Clinical Pharmacology and Therapeutics Section, Institute for Infection and Immunity, St George's, University of London, London, UK
13 Clinical Pharmacology Group, Pharmacy and Medicines Directorate, St George's University Hospitals NHS Foundation Trust, London, UK
14 Novo Nordisk Research Centre Oxford, Old Road Campus, Oxford, UK
15 British Heart Foundation Cardiovascular Epidemiology Unit, Department of Public Health and Primary Care, University of Cambridge, Cambridge, UK
16 Istituto di Ricerca Genetica e Biomedica, Consiglio Nazionale delle Ricerche (CNR), Cagliari, Italy
17 Dipartimento di Scienze Biomediche, Università degli Studi di Sassari, Sassari, Italy
18 Laboratory of Genetics, National Institute on Aging, US National Institutes of Health, Baltimore, MD, USA
19 Medical Research Council Biostatistics Unit, University of Cambridge, Cambridge, UK
20 Department of Endocrinology, Clinic of Medicine, St. Olavs hospital Trondheim University Hospital, Trondheim, Norway
21 HUNT Research Centre, Department of Public Health and Nursing, NTNU – Norwegian University of Science and Technology, Levanger, Norway
22 Department of Epidemiology Research, Statens Serum Institute, Copenhagen, Denmark
23 Department of Cardiology, Copenhagen University Hospital, Copenhagen, Denmark
24 Department of Human Genetics, University of Michigan, Ann Arbor, MI, USA
25 Clinic of Medicine, St. Olavs hospital Trondheim University Hospital, Trondheim, Norway

* These authors jointly supervised this work.

†Corresponding authors:

marta.r.moksnes@ntnu.no

ben.brumpton@ntnu.no

Supplementary Data 1: Index variants in genome-wide significant loci from meta-analysis of HUNT, MGI, SardiNIA, deCODE, Interval and DBDS

Supplementary Data 2: Descriptive statistics per study

Supplementary Data 3: Protein-altering custom content genotyped variants associated (p-value<5E-8) with iron traits in HUNT

Supplementary Data 4: Novel protein-altering variants in strong LD (D'=1 or R2>0.8) with meta-analysis index variants

Supplementary Data 5: LD Score regression: Genetic correlation (rg) ± standard error and p-values are given for each pair of iron status biomarkers. The matrix is symmetrical around the diagonal.

Supplementary Data 6: Bayesian colocalization anlaysis of iron trait loci (index variant ± 500Kb) and cis-eQTLs per tissue from GTEx v8

Supplementary Data 7: DEPICT: Top 10 tissues prioritized for trait associated loci

Supplementary Data 8: DEPICT: Genes prioritized at FDR<0.05

Supplementary Data 9: DEPICT: Top 10 gene sets prioritized per trait

Supplementary Data 10: Polygenic Priority Scores for the 1% top ranked genes for serum iron.

Supplementary Data 11: Polygenic Priority Scores for the 1% top ranked genes for serum ferritin.

Supplementary Data 12: Polygenic Priority Scores for the 1% top ranked genes for total iron binding capacity.

Supplementary Data 13: Polygenic Priority Scores for the 1% top ranked genes for transferrin saturation percentage.

Supplementary Data 14: Single variant PheWAS (phecodes)

Supplementary Data 15: Single variant PheWAS (continuous traits and biomarkers)

Supplementary Data 16: Single variant PheWAS (red blood cell related traits)

Supplementary Data 17: GRS-PheWAS (phecodes)

Supplementary Data 18: GRS-PheWAS (continuous traits and biomarkers)

Supplementary Data 19: GRS-PheWAS (red blood cell related traits)

Supplementary Data 20: Non-linear Mendelian Randomization: Effect of serum iron on all-cause mortality in HUNT per quantile of the residual iron distribution

Supplementary Data 21: Non-linear Mendelian Randomization: Effect of serum ferritin on all-cause mortality in HUNT per stratum

Supplementary Data 22: Non-linear Mendelian Randomization: Effect of Transferrin Saturation Percentage (TSP) on all-cause mortality in HUNT per quantile of the TSP residual distribution

Supplementary Data 23: Non-linear Mendelian Randomization: Effect of total iron binding capacity (TIBC) on all-cause mortality per quantile of the residual TIBC distribution
