# Supplementary material for: Genome-wide meta-analysis of iron status biomarkers and the effect of iron on all-cause mortality in HUNT
Source: Commun Biol. 2022 Jun 16;5:591. doi: 10.1038/s42003-022-03529-z (PMC9203493; doi:10.1038/s42003-022-03529-z)
Supplement: Supplementary file 4 — Reporting Summary [file 42003_2022_3529_MOESM4_ESM.pdf]

nature portfolio

Corresponding author(s): Marta R. Moksnes, Ben M. Brumpton

Last updated by author(s): May 13, 2022

Reporting Summary

Nature Portfolio wishes to improve the reproducibility of the work that we publish. This form provides structure for consistency and transparency in reporting. For further information on Nature Portfolio policies, see our Editorial Policies and the Editorial Policy Checklist.

Statistics

For all statistical analyses, confirm that the following items are present in the figure legend, table legend, main text, or Methods section.

n/a	Confirmed	
☐	☒	The exact sample size (*n*) for each experimental group/condition, given as a discrete number and unit of measurement
☒	☐	A statement on whether measurements were taken from distinct samples or whether the same sample was measured repeatedly
☐	☒	The statistical test(s) used AND whether they are one- or two-sided<br>*Only common tests should be described solely by name; describe more complex techniques in the Methods section.*
☐	☒	A description of all covariates tested
☐	☒	A description of any assumptions or corrections, such as tests of normality and adjustment for multiple comparisons
☐	☒	A full description of the statistical parameters including central tendency (e.g. means) or other basic estimates (e.g. regression coefficient) AND variation (e.g. standard deviation) or associated estimates of uncertainty (e.g. confidence intervals)
☐	☒	For null hypothesis testing, the test statistic (e.g. *F*, *t*, *r*) with confidence intervals, effect sizes, degrees of freedom and *P* value noted<br>*Give P values as exact values whenever suitable.*
☒	☐	For Bayesian analysis, information on the choice of priors and Markov chain Monte Carlo settings
☒	☐	For hierarchical and complex designs, identification of the appropriate level for tests and full reporting of outcomes
☐	☒	Estimates of effect sizes (e.g. Cohen's *d*, Pearson's *r*), indicating how they were calculated

Our web collection on statistics for biologists contains articles on many of the points above.

Software and code

Policy information about availability of computer code

Data collection

We used the following publicly available software (URLs below) to generate and analyze data:
R v.3.6.4
BOLT-LMM v.2.3.4, https://alkesgroup.broadinstitute.org/BOLT-LMM/downloads/
EMMAX, https://github.com/statgen/EPACTS
METAL, https://genome.sph.umich.edu/wiki/METAL
UCSC LiftOver (command line tool), http://genome.ucsc.edu/cgi-bin/hgLiftOver
ANNOVAR v.2019Oct24, https://annovar.openbioinformatics.org
plink v1.9, https://zzz.bwh.harvard.edu/plink/
DEPICT, https://data.broadinstitute.org/mpg/depict/
Coloc, https://chr1swallace.github.io/coloc/articles/a01_intro.html
PoPS v0.1, https://github.com/FinucaneLab/pops
GCTA, https://cnsgenomics.com/software/gcta/
LDSC, https://github.com/bulik/ldsc
SUMMnlmr, https://github.com/amymariemason/SUMnlmr

Data analysis

We used the following publicly available software (URLs below) to generate and analyze data:
R v.3.6.4
BOLT-LMM v.2.3.4, https://alkesgroup.broadinstitute.org/BOLT-LMM/downloads/
EMMAX, https://github.com/statgen/EPACTS
METAL, https://genome.sph.umich.edu/wiki/METAL
UCSC LiftOver (command line tool), http://genome.ucsc.edu/cgi-bin/hgLiftOver
ANNOVAR v.2019Oct24, https://annovar.openbioinformatics.org

plink v1.9, https://zzz.bwh.harvard.edu/plink/
DEPICT, https://data.broadinstitute.org/mpg/depict/
Coloc, https://chr1swallace.github.io/coloc/articles/a01_intro.html
PoPS v0.1, https://github.com/FinucaneLab/pops
GCTA, https://cnsgenomics.com/software/gcta/
LDSC, https://github.com/bulik/ldsc
SUMMnlmr, https://github.com/amymariemason/SUMnlmr

For manuscripts utilizing custom algorithms or software that are central to the research but not yet described in published literature, software must be made available to editors and reviewers. We strongly encourage code deposition in a community repository (e.g. GitHub). See the Nature Portfolio guidelines for submitting code & software for further information.

Data

Policy information about availability of data

All manuscripts must include a data availability statement. This statement should provide the following information, where applicable:

- Accession codes, unique identifiers, or web links for publicly available datasets
- A description of any restrictions on data availability
- For clinical datasets or third party data, please ensure that the statement adheres to our policy

The data supporting the findings are available in the Supplementary Data and upon request. The UK Biobank data can be obtained by application (ukbiobank.ac.uk). Summary level data from previously published meta-analysis in deCODE, Interval and DBDS are available from https://www.decode.com/summarydata/. Variant associations with phecodes, continuous traits and biomarkers used to generate Figure 1 are accessible from https://pheweb.org/UKB-TOPMed/ and https://pan.ukbb.broadinstitute.org/. The data underlying Figure 2 are given in Supplementary Data 20-23. The GWAS meta-analysis summary level data from the current study are available from NTNU Open Research Data, https://dataverse.no/dataverse/ntnu (DOI: https://doi.org/10.18710/S9TJEL).

Field-specific reporting

Please select the one below that is the best fit for your research. If you are not sure, read the appropriate sections before making your selection.

☒ Life sciences ☐ Behavioural & social sciences ☐ Ecological, evolutionary & environmental sciences

For a reference copy of the document with all sections, see nature.com/documents/nr-reporting-summary-flat.pdf

Life sciences study design

All studies must disclose on these points even when the disclosure is negative.

Sample size	The sample size for each cohort consisted of all individuals with genotype and phenotype information from each study after quality control.
Data exclusions	No data exclusion
Replication	Three independent cohorts from Norway (HUNT), the US (MGI) and Italy (SardiNIA) were meta-analysed with previous meta-analysis from Denmark (DBDS), Iceland (deCODE) and UK (Interval), and a we performed a test for heterogeneity between the studies.
Randomization	Not relevant, no randomization.
Blinding	Not relevant as this was a GWAS meta-analysis.

Reporting for specific materials, systems and methods

We require information from authors about some types of materials, experimental systems and methods used in many studies. Here, indicate whether each material, system or method listed is relevant to your study. If you are not sure if a list item applies to your research, read the appropriate section before selecting a response.

Materials & experimental systems

n/a	Involved in the study	
☒	☐	Antibodies
☒	☐	Eukaryotic cell lines
☒	☐	Palaeontology and archaeology
☒	☐	Animals and other organisms
☐	☒	Human research participants
☒	☐	Clinical data
☒	☐	Dual use research of concern

Methods

n/a	Involved in the study	
☒	☐	ChIP-seq
☒	☐	Flow cytometry
☒	☐	MRI-based neuroimaging

Human research participants

Policy information about studies involving human research participants

Population characteristics	Study participants from Norway<br>The Norwegian data include participants from the HUNT study, which is a population-based health study including ~123 000 participants (aged 20 years or older), of which about 70 000 have been genotyped.<br><br>Study participants from the US<br>The US data included participants from the Michigan Genomics Initiative: Approximately 80 000 articipants (aged 18 years or older) have predominantly been enrolled prior to surgical procedures, of which over 59 000 individuals have been genotyped.<br><br>Study participants from Italy<br>The Italian data included participants from the SardiNIA study, which is a population-based health study including 6602 individuals from SardiNIA. The majority of the participants have been genotyped.
Recruitment	See above.
Ethics oversight	All study participants have given informed consent. The analyses in HUNT has approval from the Norwegian Data Protection Authority and the Regional Ethics Committee for Medical and Health Research Ethics in Central Norway (REK Reference Number: 2014/144), the analyses in MGI are approved by the Institutional Review Board of the University of Michigan Medical School (IRB Reference Number: HUM00094409), the analyses in SardiNIA are approved by the local ethics committee for the Istituto di Ricerca Genetica e Biomedica-CNR (IRGB-CNR; Cagliari, Italy), and the analyses in UK Biobank are covered by the ethics approval for UK Biobank studies (application 24460) from the NHS National Research Ethics Service on 17th June 2011 (Ref 11/NW/0382) and extended on 10th May 2016 (Ref 16/NW/0274).

Note that full information on the approval of the study protocol must also be provided in the manuscript.
